# Headaches and facial pain attributed to SARS‐CoV‐2 infection and vaccination: a systematic review

**DOI:** 10.1111/ene.16251

**Published:** 2024-02-28

**Authors:** Dimos‐Dimtirios D. Mitsikostas, Edoardo Caronna, Marina De Tommaso, Christina I. Deligianni, Esme Ekizoglu, Hayrunnisa Bolay, Carl H. Göbel, Espen Saxhaug Kristoffersen, Christian Lampl, Elena Moro, Patricia Pozo‐Rosich, Johann Sellner, Gisela Terwindt, Pablo Irimia‐Sieira

**Affiliations:** ^1^ Neurology Department, Aeginition Hospital, Medical School National & Kapodistrian University of Athens Athens Greece; ^2^ Headache Unit, Neurology Department Hospital Universitari Vall d'Hebron Barcelona Spain; ^3^ Headache and Neurological Pain Research Group, Department of Medicine Vall d'Hebron Research Institute, Universitat Autònoma de Barcelona Barcelona Spain; ^4^ Neurophysiopathology Unit, DiBrain Department Aldo Moro University Bari Italy; ^5^ Athens Naval Hospital Athens Greece; ^6^ Department of Neurology, Faculty of Medicine Istanbul University, Istanbul Istanbul Turkey; ^7^ Department of Neurology and Algology, NÖROM Gazi University Ankara Ankara Turkey; ^8^ Department of Neurology University Hospital Schleswig‐Holstein Lübeck Germany; ^9^ Department of Neurology Akershus University Hospital Lørenskog Norway; ^10^ NorHEAD, Norwegian Centre for Headache Research Akershus University Hospital Lørenskog Norway; ^11^ Department of General Practice, HELSAM University of Oslo Oslo Norway; ^12^ Department of Neurology and Stroke Unit Konventhospital Barmherzige Brüder Linz Linz Austria; ^13^ Headache Medical Center Linz Linz Austria; ^14^ Division of Neurology, CHU of Grenoble, Grenoble Institute of Neurosciences, Grenoble Alpes University Grenoble France; ^15^ Department of Neurology Landesklinikum Mistelbach‐Gänserndorf Mistelbach Austria; ^16^ Department of Neurology Leiden University Medical Center Leiden The Netherlands; ^17^ Neurology Department Clinica Universidad de Navarra Pamplona Spain

**Keywords:** chronic daily headache, headache, neurological disorders

## Abstract

**Background and purpose:**

The aim was to provide insights to the characteristics of headache in the context of COVID‐19 on behalf of the Headache Scientific Panel and the Neuro‐COVID‐19 Task Force of the European Academy of Neurology (EAN) and the European Headache Federation (EHF).

**Methods:**

Following the Delphi method the Task Force identified six relevant questions and then conducted a systematic literature review to provide evidence‐based answers and suggest specific diagnostic criteria.

**Results:**

No data for facial pain were identified in the literature search. (1) Headache incidence during acute COVID‐19 varies considerably, with higher prevalence rates in prospective compared to retrospective studies (28.9%–74.6% vs. 6.5%–34.0%). (2) Acute COVID‐19 headache is usually bilateral or holocranial and often moderate to severe with throbbing pain quality lasting 2–14 days after first signs of COVID‐19; photo‐phonophobia, nausea, anosmia and ageusia are common associated features; persistent headache shares similar clinical characteristics. (3) Acute COVID‐19 headache is presumably caused by immune‐mediated mechanisms that activate the trigeminovascular system. (4) Headache occurs in 13.3%–76.9% following SARS‐CoV‐2 vaccination and occurs more often amongst women with a pre‐existing primary headache; the risk of developing headache is higher with the adenoviral‐vector‐type vaccines than with other preparations. (5) Headache related to SARS‐CoV‐2 vaccination is mostly bilateral, and throbbing, pressing, jolting or stabbing. (6) No studies have been conducted investigating the underlying mechanism of headache attributed to SARS‐CoV‐2 vaccines.

**Conclusion:**

The results of this joint EAN/EHF initiative provide a framework for a better understanding of headache in the context of SARS‐CoV‐2 infection and vaccination.

## INTRODUCTION

Several neurological complications are emerging of the coronavirus disease 2019 (COVID‐19) caused by the severe acute respiratory syndrome coronavirus 2 (SARS‐CoV‐2) [1]. Like other coronaviruses, SARS‐CoV‐2 targets the human respiratory system primarily but it can also lead to nervous system dysfunction [2, 3, 4]. Headache is experienced as a presenting symptom in COVID‐19 and as an adverse event (AE) of the SARS‐CoV‐2 vaccines, as well as a key symptom in the condition that occasionally follows the resolution of acute COVID‐19, the so‐called post‐COVID‐19 condition [5, 6, 7]. According to the World Health Organization, the post‐COVID‐19 condition is defined as the continuation or development of new symptoms 3 months after the initial SARS‐CoV‐2 infection, with these symptoms lasting for at least 2 months with no other explanation [8]. However, the specific characteristics of this secondary headache type have not been fully identified. Furthermore, the causal relationship and origin of the headache remain questionable, for example whether it contributes to anatomical central nervous system (CNS) lesions, to regional or to general inflammation responses. The Headache Scientific Panel of the European Academy of Neurology (EAN), along with the European Headache Federation (EHF) appointed a Task Force to provide evidence‐based information on the prevalence and the specific features of the headache and/or facial pain attributed to acute COVID‐19, attributed to the phase after the resolution of acute COVID‐19 and to SARS‐CoV‐2 vaccines, and to propose clinical diagnostic criteria for each headache type. These headaches require recognition in the International Classification of Headache Disorders (ICHD) more specifically than code 9.2.2 (headache attributed to viral systemic infection) listed in the current version 3 [9], because the incidence of COVID‐19, a condition with greatly increased mortality rates, remains very high, being amongst the most common viral infections worldwide.

## METHODS

The Task Force appointed by the Headache Scientific Panel co‐chairs, along with the Neuro‐COVID‐19 Task Force of EAN. The President of the EHF was invited as the EHF representative. Following the Delphi method [10] the Task Force had several virtual meetings (DDM served as facilitator) to establish the specific questions that should be addressed, to interpret the results of the literature search and to discuss the findings and provide evidence‐based answers to the established questions.

### Clinically relevant questions that should be addressed

Amongst several questions proposed by the panellists the Task Force selected six main questions that should be answered according to the PICO model (P population, I intervention, C control, O outcome) in three Delphi rounds and thereafter a literature search was performed.

### Systematic literature search

The systematic review was guided by the Preferred Reporting Items for Systematic Reviews and Meta‐Analyses (PRISMA) [11]. PubMed, MEDLINE, Embase, Web of Sciences database and the Cochrane COVID‐19 Study Registry were used for the literature search. Studies were restricted to those published from December 2019 (beginning of the outbreak of COVID‐19) to the date of the search operation. Types of study eligible for inclusion in the analysis included original articles and cross‐sectional and cohort studies written in English. Case series and case reports, conference abstracts, reviews, editorials, comments, expert opinions, preprints, unpublished data, studies involving an analysis of mixed patient results, placebo‐controlled phase‐1 and ‐2 trials, vaccine studies including less than 1000 subjects, studies with specific concomitant conditions and studies with paediatric participants (<18 years old) were excluded. The search strategy was (‘COVID‐19’ OR ‘COVID19’ OR ‘coronavirus’ OR ‘SARS‐CoV‐2’ OR ‘SARS‐CoV2’) AND (‘headache’ OR ‘facial pain’ OR ‘neuralgia’). All studies conducted in primary, secondary and tertiary care settings with no restrictions on location of healthcare setting were included, for example observational cohort studies, randomized controlled studies and cross‐sectional studies. The main outcomes included features of headache attributed to COVID‐19, the condition after acute COVID‐19 and the condition after SARS‐CoV‐2 vaccination, according to the PICO model [12, 13].

The study selection followed a two‐stage process. First, titles and/or abstracts of studies were screened by three independent reviewers for each specific PICO and the full text of selected articles was assessed for eligibility by three reviewers independently, whilst any disagreement was resolved through further discussion. The final number of included/excluded publications is presented in a PRISMA flow chart for each PICO and all data were independently extracted by three authors (EE, EC and CD) using a data extraction form. A data quality strategy was designed based on the Checklist for Cohort Studies guidelines to guarantee the integrity of the extracted data. Two independent reviewers assessed the quality of the studies, and any discrepancies were discussed by a third reviewer. The findings are presented in narrative form including tables and figures to aid in data presentation, where appropriate. All the data were categorized as per the study, the authors, country, publication date, study design, setting, characteristics and demographics of cases, risk factors, underlying disease/s, name of SARS‐CoV‐2 vaccine, doses of vaccine, time to symptoms from vaccine administration, clinical presentation, severity of infection, diagnostic studies, imaging, complications, time to normal and outcome. A descriptive and qualitative synthesis of data was performed. A quantitative synthesis was performed in Microsoft Excel and was assessed using R software. Two independent contributors interpreted the data and any discrepancies were resolved by discussion with another reviewer, if needed. Analysis of the following subgroups or subsets was carried out: COVID‐19 group, condition after the resolution of the acute COVID‐19 group, and SARS‐CoV‐2 vaccination group.

### Data interpretation and evidence‐based answering

Based on the data collected from each subgroup, the answers to the specific PICOs were constructed. One panellist was responsible for answering each PICO (Q1, PP‐R; Q2, HB; Q3, MDT; Q4, CL; Q5, CG; Q6, DDM) and submitted it to the facilitator separately. Based on these reports the facilitator prepared the first draft that was reviewed and discussed by all panellists in four rounds. After completing the evidence‐based answers the facilitator prepared a draft for the diagnostic criteria for headache attributable to SARS‐CoV‐2 infection and vaccine, which was reviewed and discussed in two additional rounds. All panellists reviewed and edited the final manuscript in several rounds to achieve a final consensus for all answers and proposed diagnostic criteria.

## RESULTS

The Task Force selected six clinically relevant questions that should be addressed [10]:

Q1. Is headache and/or facial pain a common symptom of acute COVID‐19 and/or after recovery?
Q2. What are the clinical characteristics of headache and/or facial pain attributed to acute COVID‐19 and/or after recovery?
Q3. How is headache and/or facial pain triggered during COVID‐19 and/or after recovery?
Q4. Is SARS‐CoV‐2 vaccination associated with headache/facial pain, and is there a distinction between different types of vaccines?
Q5. What are the clinical characteristics of headache and/or facial pain attributed to SARS‐CoV‐2 vaccination?
Q6. How is headache and/or facial pain triggered by different SARS‐CoV‐2 vaccines?

**FIGURE 1:**
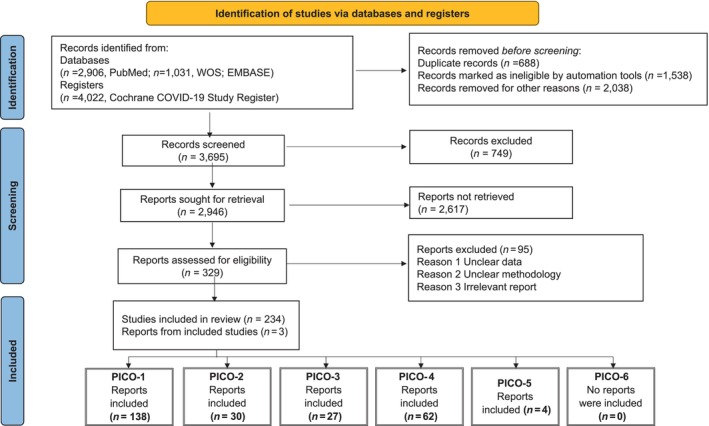
The PRISMA flow diagram of the literature search to answer the six questions (PICOs). Several references are common between different PICOs, while 3 references were included in the anaylisis after reviewing the reference lists of the articles, although they did not appear in the initiale literature search.

In Figure 1 the PRISMA charts for each search are presented. The list of references included in the review to answer each PICO is presented in six separate appendices.

### Q1. Is headache and/or facial pain a common symptom of COVID‐19 and/or after acute COVID‐19?

From the search performed, 138 articles were included to answer this PICO (Appendix S1). Headache prevalence in the acute phase of COVID‐19 is highly variable amongst studies. Most studies were conducted during the first wave of the pandemic, when the native strain of SARS‐CoV‐2 was the one circulating. In this setting, prevalence rates are remarkably affected by study design: retrospective studies generally report far lower proportions of headache (6.5%–34%) [14, 15, 16, 17], whilst cross‐sectional or prospective studies describe a prevalence ranging from 28.9% up to 74.6% [18, 19]. National/international registries specifically designed to collect neurological symptoms report lower rates [20, 21, 22] than those in headache‐focused studies [19, 23, 24]. Most of the studies from the first wave focused on inpatients; however, for outpatients headache prevalence seems to be higher (52% inpatients vs. 70% outpatients) [25]. Geographical differences in headache prevalence have been observed, with India reporting the lowest rates [26]. Data on headache prevalence according to other SARS‐CoV‐2 variants are scarce. In unvaccinated inpatients, the Delta variant of SARS‐CoV‐2 is associated with higher proportions of headache in the acute phase (32.7%) compared with Wuhan (20.9%) and Alpha (11.8%) [27]. Another study reported higher headache prevalence for Delta compared with Alpha [28]. The proportion of headache during Omicron infection is still to be clarified, as current data are scarce [29]. Not only the change in SARS‐CoV‐2 variants but also the introduction of COVID‐19 vaccines has influenced headache prevalence in the acute phase of COVID‐19. Although studies are limited, vaccinated people report less headache than those unvaccinated [30]. Several studies describe headache being more frequent in women [19, 23, 31, 32, 33], at a younger age [19, 34, 35] and in patients with personal history of migraine [23, 36, 37]. Concerning ethnicity, Hispanic people seem to report headache more frequently [38], although more data are needed.

Headache prevalence in the phase after the resolution of acute COVID‐19 is more consistent across studies, because of more similar methodologies. Prevalence slightly changes according to the time point: at 3 months 19%–38% [39, 40], 6 months 10%–16.8% [39, 41], 9 months 16%–26.8% [39, 42], 12–15 months 9%–17.3% [43, 44]. In specialized outpatient post‐COVID clinics, headache prevalence reaches 71% [45]. However, study cohorts mainly include people with COVID‐19 in 2020 and data on other than SARS‐CoV‐2 Alpha variants are therefore lacking. In unvaccinated inpatients, after 6 months from disease onset, headache prevalence was higher in individuals infected with the Delta variant (12.9%) compared to the native one (5.5%) or Alpha (3.8%) [27]. One study compared headache in the post COVID‐19 condition according to vaccination status, with lower rates observed in vaccinated individuals (14.0% vaccinated vs. 21.8% non‐vaccinated) [46]. Some studies describe headache being more frequent in women [39, 47]. Association between headache and age is unclear, due to limited specific analysis [39]. Studies suggest that personal history of migraine [37, 39] is not associated with persisting headache, but this finding is still controversial [36]. No conclusion can be obtained on headache prevalence during the phase after recovery from acute COVID‐19 in relation to anxiety/depression, due to lack of data.

No formal conclusion can be drawn on facial pain prevalence, due to lack of data.

#### Comment

Retrieving one pooled prevalence for headache in COVID‐19 can be misleading, as headache prevalence rates should be adjusted to each specific setting including SARS‐CoV‐2 strain and vaccination status and should consider the methodology used for its estimation. Usefulness of neurological registries to estimate headache prevalence may be affected by willingness to report headache compared to other neurological conditions considered more severe. In specialized outpatient post‐COVID clinics, headache is remarkably common, which prompts the treating physicians to have knowledge on this matter. Since in 2023 the COVID‐19 scenario has changed compared to the beginning of the pandemic, it is believed that there are questions of interest that need to be further investigated, for example headache incidence as an AE of different SARS‐CoV‐2 vaccines, with ethnicity, SARS‐CoV‐2 variants, individual medical history and the severity of COVID‐19 as variables, the latter because there is some evidence that the presence of headache during acute COVID‐19 may count as a marker of good COVID‐19 prognosis [48].

#### Conclusion

The incidence of headache attributed to acute COVID‐19 varies considerably amongst surveys (6.5%–74.6%), by study design, speciality of investigators, the population included in the survey, gender (more frequent in women), age (more frequent in younger age), pre‐existing migraine (more frequent in those with migraine) and vaccination status (vaccinated people report less headache than those unvaccinated), amongst others. After acute COVID‐19 the headache prevalence varies by the time lag after first symptoms of COVID‐19 (decreased prevalence with increasing time after infection) and as in COVID‐19 by the interest of investigators (e.g., headache specialists report higher rates than non‐headache specialists), gender and vaccination status. There are no data for facial pain prevalence in COVID‐19 or following resolution of the acute phase of COVID‐19.

### Q2. What are the clinical characteristics of headache and/or facial pain attributed to acute COVID‐19 and/or after COVID‐19?

From the search performed, 30 articles were included (Appendix S2). Only headache‐focused studies from the first wave of the pandemic had available information on headache characteristics. In the acute phase of COVID‐19, different localizations, pain qualities and severity have been described. Headache is more commonly bilateral/holocranial (42%–94%) [22, 34]. It is moderate–severe in 15%–88% [23, 34] and throbbing in 11%–64% [23, 49]. The most frequent associated symptoms include migraine features such as photo‐phonophobia in 22%–63% [23, 49], nausea in 24%–32% [22, 23] and worsening with movement in 14%–53% [22, 50]. Two phenotypes may eventually be distinguished: migraine‐like in up to 51% [22] and tension‐type‐like in up to 43% [51]. The median duration of headache in inpatients during the first wave of the pandemic was 7–14 days [19, 39, 50, 51]. Response to acute treatment (most often with paracetamol) has been reported as unsatisfactory in 21%–41% [19, 52]. In other reports, 46% of participants rated aspirin as effective in headache relief, followed by ibuprofen (17%) and paracetamol (14%) [53]. One study showed that at least one headache red flag is reported in up to 95% of patients [54]. Anosmia and ageusia are significantly associated with headache attributed to acute COVID‐19 [19, 22, 35]. No data on the influence of different SARS‐CoV‐2 variants and vaccination status on headache characteristics are available.

After resolution of the acute phase of COVID‐19, similar headache characteristics have been reported. According to the study, the migraine‐like (up to 74%) [55] or the tension‐type‐like phenotype (up to 90%) [27] is the most common; however, data on headache characteristics are very limited. Descriptions on the temporal pattern and causal relationship of persistent headache in the phase after the resolution of acute COVID‐19 are lacking. No data exist for facial pain.

#### Comment

Most of the questions that are supposed to be the most relevant ones currently are unanswered, for example the headache characteristics in vaccinated compared to non‐vaccinated individuals; in several SARS‐CoV‐2 variants the clinical characteristics in the long term are missing etc.

#### Conclusion

Headache attributed to COVID‐19 is moderate to severe, throbbing and bilateral/holocranial, accompanied by photo‐phonophobia, nausea, anosmia and ageusia, and it worsens with movement, most often lasting 7–14 days after the initial symptoms of SARS‐CoV‐2 infection (Table 1). Headache after recovery from COVID‐19 shares similar characteristics as in the acute phase. No data exist for facial pain.

**TABLE 1:** Headache attributed to SARS‐CοV‐2 characteristics.

Headache characteristics	Frequency observed (%)	References
Migraine‐like	Up to 51	22
Tension‐type‐like	Up to 43	50
Bilateral/holocranial location	42–92	22, 34
Moderate to severe intensity	15–88	22, 34
Throbbing quality	11–64	23, 49
With photo‐phonophobia	22–63	23, 49
With nausea	24–32	22, 23
With worsening with movement	14–53	22, 50
With response to aspirin, ibuprofen or paracetamol	46, 17, 14 respectively	52

### Q3. How is headache and/or facial pain triggered during COVID‐19 and/or after acute COVID‐19?

From the search performed, 27 articles were identified to answer this PICO (Appendix S3). Since the first cases of COVID‐19 with neurological involvement were described, the presence of biomarkers of axonal degeneration in the cerebrospinal fluid (CSF), as neurofilaments, suggested potential viral tropism to the CNS. However, the very limited number of cases with detectable viral RNA in CSF as well as the few findings of inflammatory signs probably suggest that SARS‐CoV‐2 does not cross the blood–brain barrier (BBB), replicate in neurons or persist in the brain [56]. In the first pandemic wave, two main mechanisms were considered as potential mechanisms for headache: activation of the trigeminovascular system (TVS) by systemic inflammation, or direct involvement of SARS‐CoV‐2 within the peripheral sites of the TVS via retrograde access through olfactory mucosa, a hypothesis supported by concomitant anosmia [19, 57, 58, 59]. Evidence for the latter hypothesis has not been found so far. Others found that headache in people with COVID‐19 is associated with intracranial hypertension in the absence of meningitic or encephalitic features and suggested that coagulopathy associated with COVID‐19 leading to intracranial hypertension could be an explanation for headache [60]. Generally, there is no good documentation for any pathogenesis for headache in the context of COVID‐19.

#### Direct viral entry through olfactory mucosa

Studies found that headache is associated with acute rhinosinusitis activating the TVS and the nociceptive pathways, leading to a migraine‐like mechanism, but most probably robust inflammation in the systemic compartment may activate the TVS [61, 62]. Several surveys confirmed that people with COVID‐19 who reported headache also experienced anosmia and ageusia, suggesting that SARS‐CoV‐2 may activate the peripheral sites of the TVS [19, 35, 57, 58, 59, 63].

Because these people with anosmia, ageusia and headache also had a lower risk of mortality and fewer serum biomarkers suggestive of cytokine storm, one could indirectly point to viral nasal mucosa tropism as a potential cause of headache during COVID‐19 [35, 64]. Limited inflammatory responses have been identified either in endothelial cells [65] or within the CSF [66, 67], further supporting the hypothesis of a direct TVS activation by systemic inflammation.

#### Inflammatory and cytokine storm, endothelial cell damage and altered state of BBB function

Several studies in humans, animals and in vitro models indicated the pivotal role of inflammation in COVID‐19 headache, with cytokine storm and BBB damage [68]. In particular, SARS‐CoV‐2 spike proteins may induce the release of proinflammatory mediators and damage brain endothelial cells with the consequence of an altered state of BBB function [69]. In addition, a direct effect of SARS‐CoV‐2 on microglia might trigger proinflammatory responses, followed by cyto‐pathic effects within the CNS [70]. Because SARS‐CoV‐2 contains amyloid‐forming sequences, it has been suggested that amyloid aggregates might induce inflammatory responses leading to TVS activation and headache, but this has not been confirmed [71, 72].

Stronger innate immune system activation and circulating proinflammatory molecules were also shown in COVID‐19 patients with severe headache. Circulating levels of HMGB1 (a nuclear protein that binds to DNA and acts as an architectural chromatin‐binding factor) and NLRP3 (a protein expressed in macrophages that detects products of damaged cells such as extracellular ATP) were correlated with headache response to paracetamol and hospital stay in COVID‐19 patients [73].

#### Increased CSF pressure because of cerebral venous thrombosis

People with headache during acute COVID‐19 frequently exhibit increases of D‐dimer levels, coagulation abnormalities and elevated markers of inflammation in peripheral blood. Thus, an immune‐mediated and vascular pathogenesis for headache has been discussed [18, 35, 50]. Potentially immune‐mediated headache attributed to CNS complications of COVID‐19, for example cerebral venous thrombosis, coma and encephalitis, have been described as well [74]. Headache attributed to cerebral thrombosis in the context of COVID‐19 is intractable and usually accompanied by focal neurological symptoms and signs [75].

#### Headache in the phase after the resolution of acute COVID‐19

A fifth of people with headache in COVID‐19 develop a chronic pattern of headache [76]. People with post‐COVID‐19 symptoms including headache had higher baseline proinflammatory, chemotactic and angiogenic cytokines [77]. Signs of neuro‐inflammation and neuroaxonal and glial degeneration, measured with neurofilament light chain and glial fibrillary astrocytic protein, were lower in the blood of people with persistent headache in the phase after the resolution of acute COVID‐19 compared to people with severe COVID‐19, indicating no neuroaxonal and glial damage or reactive gliosis in people with long‐term persistent headache after COVID‐19 [78]. Others found that pre‐existing migraine was not associated with chronic headache in the phase after the resolution of acute COVID‐19, but it was a risk factor for other persistent symptoms, for example fatigue, indicating that pre‐existing migrainous mechanisms might lead to the development of systemic symptoms in the phase after the resolution of acute COVID‐19 [79]. Furthermore, SARS‐CoV‐2 itself or the associated inflammation might activate the TVS or induce persistent endothelial dysfunction which could lead to persistent chronic headache, as well [80, 81].

#### Conclusion

There is no univocal hypothesis about the mechanism of headache during acute COVID‐19 or post‐COVID‐19 condition. Headache, frequently isolated or associated with few other COVID‐19 symptoms, might be induced by proinflammatory cytokine induced activation of the TVS. Cytokine storm, with endothelium disruption and BBB damage, possible microglia, and amyloid cascade activation, might be the cause of a more severe acute COVID‐19 syndrome including headache as an accompanying symptom. Potential mechanisms of pre‐existent primary headache, along with indirect activation of the TVS and persistent inflammation, catabolism syndrome and endothelial dysfunction, have been suggested as possible pathophysiological mechanisms of persistent headache during the phase after the resolution of acute COVID‐19. Facial pain or even headache may be related partially to protective masks during COVID‐19 as well [82, 83].

### Q4. Is SARS‐CoV‐2 vaccination associated with headache/facial pain, and is there a distinction between different types of vaccines?

Sixty‐two articles were identified (Appendix S4). According to the Vaccine Adverse Events Reporting System, headache was the fifth most reported AE post‐vaccination after administration of any SARS‐CoV‐2 vaccine [84]. Notably, headache is a frequent AE after any vaccination [85]. Headache as a very common AE category in SARS‐CoV‐2 vaccines (frequency of occurrence ≥1/10) was reported in the mean by 53% of the vaccine recipients. Headache is observed in 13.3%–76.9% following SARS‐CoV‐2 vaccines and mostly experienced by females with pre‐existing primary headaches. The highest frequency of headache was reported with the adenoviral vector type (AstraZeneca, J&J, Sputnik V 36%–72%), followed by mRNA vaccines (BioNTech/Pfizer, Moderna 29%–55%) and then the inactivated type (Sinopharm 17%–35%). Female sex and thyroid disease were significantly associated with headache attributed to SARS‐CoV‐2 vaccines. The number of reported AEs was significantly higher amongst females with pre‐existing primary headaches and higher education; the reactogenicity of the vaccine amongst individuals between 18 and 65 years was higher than individuals above 65 years. Headache was observed in 33% of all people after the first vaccination and 59% after the second vaccination [86]. A rare secondary headache attributed to cerebral venous thrombosis (in most of the cases vaccine‐associated immune thrombosis and thrombocytopenia) occurs 5–30 days after SARS‐CoV‐2 vaccination [87].

Other common neurological AEs to SARS‐CoV‐2 vaccines include facial palsy, intracerebral haemorrhage and Guillain–Barré syndrome [88].

#### Comment

The AEs reported in relation to SARS‐CoV‐2 vaccines may not be a true representation because neurological AEs like headaches are a common occurrence with all vaccinations. Curation of large‐scale electronic health record documented frequencies of AEs are much lower than their solicited frequencies reported in clinical trials (e.g., BioNTech/Pfizer 1%; Moderna 1.1%). In addition, headache was recorded frequently as an AE in the placebo arms of randomized clinical trials for SARS‐CoV‐2 vaccines indicating a nocebo response [89, 90].

#### Conclusion

Headache is observed in 13.3%–76.9% of people after SARS‐CoV‐2 vaccination and mostly experienced by females with pre‐existing primary headaches. The highest frequency of headache was reported with the adenoviral vector type, followed by mRNA vaccines and then the inactivated type. The incidence of headache varied by the order of vaccine administration, being higher in the second dose, and was related to a cytokine storm most probably, apart from rare cases of secondary headache attributed to cerebral venous thrombosis. For people presenting with severe, drug‐resistant and delayed‐onset post‐SARS‐CoV‐2 vaccination headache, it could represent a possible sign of cerebral venous thrombosis which is most often attributed to thrombocytopenia and requires special management [87, 91]. No data for facial pain are available.

### Q5. What are the clinical characteristics of headache and/or facial pain attributed to SARS‐CoV‐2 vaccination?

Out of 19 screened articles focused on the clinical characteristics of headache/facial pain attributed to SARS‐CoV‐2 vaccines only four reported data on our outcome of interest and were included in this review (Appendix S5) [53, 92, 93, 94].

Three of these studies were observational, multicentre, headache‐focused and prospective and one was retrospective [92]. The first study was an observational, cross‐sectional, web‐based questionnaire study and it was conducted investigating headache after SARS‐CoV‐2 vaccination; its features included 1819 healthcare personnel (mean age 44.4 ± 13.4 years; 67.2% females) receiving inactivated virus vaccine (Sinovac), of which 93.1% received both doses. Headache occurred in 30.6% of the participants, and the headache onset was 1.8 ± 3.5 days (median 1; interquartile range 0–2 days) after vaccination. A percentage of 43.9% experienced headache after the first dose and 25% after the second dose, whereas 31.1% experienced headache following both doses. Headache duration was >3 days in 25.9%, <3 days in 74.1% of participants, <12 h in 46.6% and 12–24 h in 15.5%. Headache location was mostly bilateral, and its characteristics were throbbing (40.1%), pressing (30.4%), jolting (59.0%), stabbing (9.5%). Female gender, positive history of pre‐existing primary headaches (tension‐type headache [TTH], migraine), thyroid diseases, post‐influenza vaccination headache and COVID‐19 headache and fever were statistically significant variables of headache after SARS‐CoV‐2 vaccination [92]. The second study [93] included 2464 participants who reported headaches after ChAdOx1 nCoV‐19 (AZD1222) vaccination and 2349 participants after BNT162b2 mRNA SARS‐CoV‐2 vaccine. From these populations, the majority did not report any headache after previous vaccinations (7.4% and 9.7%, respectively, experienced post‐vaccination headaches following previous vaccinations; 90.2% and 84.9% of them reported a different type of headache compared to the headache that occurred after SARS‐CoV‐2 vaccination). Approximately 65% of both groups had a history of prior existing primary headaches and 3.7%–13% of mental disorders [53, 93]. Other most common comorbidities were hormonal, such as diseases of the thyroid or pituitary gland and the pancreas (7.7%), pulmonary (6.2%) and vascular disorders (5.0%) [53]. Most participants described monophasic events of headache [53, 93]. The average time to onset of headache was 14.5 ± 21.6 h [93] and 18.0 ± 27.0 h, respectively, and >50% presented headache after <10 h and 80% within 24 h after the vaccination; less than ~10% of the participants reported headache onset >2 days post‐vaccination [53, 93]. The average headache duration was 16.3 ± 30.4 h [2] and 14.2 ± 21.4 h [53]. The location of headache was mainly bilateral (~75%), located on the forehead (~39%), temples (~31.7%) and occipital region (~24%); the character of headache was pressing (~50%), dull pain (~39%), pulsating (18%), throbbing (16.28%). A percentage of 56.7%–60.6% of participants reported no radiation of pain [53, 93]. In the ChAdOx1 nCoV‐19 vaccinated group headache intensity was mostly severe (38.7%), followed by moderate (35.2%) or very severe (15.5%), whereas in the BNT162b2 mRNA SARS‐CoV‐2 vaccinated population pain intensity was moderate (46.2%) followed by severe (32.1%) or very severe (8.2%). Fatigue (38.8%–44.8%) and exhaustion (25.7%–34.9%) were the most common accompanying symptoms amongst subjects vaccinated with either vaccine, whilst chills (36.1%) and fever (30.4%) were described as accompanying symptoms in the ChAdOx1 nCoV‐19 vaccinated group [93] and muscle pain (23.4%) in the BNT162b2 mRNA SARS‐CoV‐2 vaccinated group [53]. Regarding migraine‐like accompanying symptoms the most frequently described were sensitivity to noise (27.8%–33.4%), sensitivity to light (26.8%–31.8%) and nausea (23.9%–28.1%), being more prevalent following the ChAdOx1 nCoV‐19 vaccination. Participants with a history of migraine reported higher duration and intensity of headache in both groups of vaccines, whilst those suffering from TTH reported higher intensity after ChAdOx1 nCoV‐19 vaccination and after BNT162b2 mRNA SARS‐CoV‐2 vaccination reported shorter latency between vaccination and pain onset, compared to those with no history of primary headaches. There was no difference in relation to time to onset or duration of headache [93], whilst females reported higher intensity in comparison with males [53]. Regarding age as a variable, there was a difference in intensity with more severe headaches in ages <55 years [93]. The above major findings are confirmed, in general, by another study conducted in a population with new onset or worsening headache within 16 days after SARS‐CoV‐2 vaccination that were admitted to the emergency department and/or hospitalized [93]. Regarding facial pain, no data are available in relation to SARS‐CoV‐2 vaccination.

#### Conclusion

Headache attributed to SARS‐CoV‐2 vaccines is usually bilateral, throbbing, pressing, jolting or stabbing. As in COVID‐19, female gender, pre‐existing primary headaches, history of thyroid disease, mental disorder, previous post‐influenza vaccination headache, occurrence of headache and/or fever in COVID‐19 increase the risk of headache after SARS‐CoV‐2 vaccination. Headache characteristics and accompanying symptoms might vary by the type of SARS‐CoV‐2 vaccine, being higher after the adenoviral‐vector‐type vaccines. No data are available in relation to SARS‐CoV‐2 vaccination.

### Q6. How is headache and/or facial pain triggered by different SARS‐CoV‐2 vaccines?

Our systematic search, which focused on the mechanism by which headache and/or facial pain is triggered by SARS‐CoV‐2 vaccines and whether all vaccines share the same mechanism for triggering headache yielded no relevant articles providing data for the outcome of interest. Only speculations under the mechanism have been reported.

In one study [] the authors concluded that migraine‐like characteristics that were reported by people with new onset or worsening headache within 16 days following vaccination could be a result of the TVS pathway activation by all the cytokines and other proinflammatory molecules released. Nevertheless, no data supporting this assumption are provided. The same assumption is supported by another study [53]. But it still remains unclear whether the intracellular S‐glycoprotein of SARS‐CoV‐2 produced by the gene supplied by the vaccine, or further immune responses triggered by the S‐glucoprotein are responsible for triggering headache and its accompanying symptoms such as chills and fever, or whether other substances (e.g., nitric oxide, cytokines, prostaglandins, substance P) might cause these symptoms.

#### Conclusion

No studies have been conducted so far to investigate the underlying mechanism of headache and/or facial pain triggered upon SARS‐CoV‐2 vaccination. Only speculations have been reported, for example that the vaccines induce the release of cytokines and other proinflammatory molecules that activate TVS leading to headache.

## CONCLUSIONS AND PROPOSAL FOR DIAGNOSTIC CRITERIA

This EAN/EHC Task Force after systematically reviewing the literature identified that the incidence of headache attributed to COVID‐19 varies considerably amongst surveys, affected by study design and settings, for example cross‐sectional or prospective studies report higher prevalence rates (28.9%–74.6%) than retrospective studies (6.5%–34%). Headache is moderate to severe, throbbing, and bilateral or holocranial, accompanied by photo‐phonophobia, nausea, anosmia, ageusia, and it worsens with movement, most often lasting 7–14 days after acute COVID‐19. Headache shares similar characteristics when present in the phase after the resolution of acute COVID‐19. Regarding the pathophysiology of headache, there is only indirect evidence that mild headache might be induced by activation of the TVS. Elevated proinflammatory cytokines in the circulation and/or in the trigeminal nociceptive matrix along with endothelium disruption and BBB damage might result in a more severe acute COVID‐19 with severe headache. Furthermore, headache occurs as an AE of SARS‐CoV‐2 vaccines in 13.3%–76.9% of vaccinated people. Headache related to SARS‐CoV‐2 vaccines is mostly bilateral, throbbing, pressing, jolting or stabbing. As in the case of headache attributed to COVID‐19, headache after SARS‐CoV‐2 vaccines is more prevalent in women, in those who had pre‐existing primary headaches, thyroid diseases, previous post‐influenza vaccination headache, COVID‐19 headache and fever during COVID‐19. The incidence of headache after SARS‐CoV‐2 vaccination varies by the type of vaccination, being higher after the adenoviral‐vector‐type vaccines. However, headache is a common AE after all vaccines. Thus, there is no good evidence that headache attributed to SARS‐CoV‐2 vaccines is a separate identity. There are no studies aimed to investigate the underlying mechanism of headache attributed to COVID‐19, the phase after the resolution of acute COVID‐19 and to SARS‐CoV‐2 vaccines, only indirect evidence. Notably, headache was often recorded as an AE in the placebo arms of trials with SARS‐CoV‐2 vaccines, indicating an increased nocebo effect. No evidence was found for the prevalence of facial pain attributable to COVID‐19, to the phase after resolution of acute COVID‐19 or to SARS‐CoV‐2 vaccines.

Based on these findings the Task Force suggests the following phenomenological criteria only for headache attributed to COVID‐19, based on the scarce available literature for some of the PICOs. According to the ICHD‐3 [9], a headache is considered as secondary when it first occurs in close temporal relation to another disorder which is known to cause headache or fulfils other criteria for causation by that disorder. In the case of headache attributed to SARS‐CoV‐2 infection, it is unclear whether it represents a secondary headache type or a worsening of a pre‐existing primary headache. The Task Force followed the format of the diagnostic criteria for secondary headaches. No diagnostic criteria for headache attributed to SARS‐CoV‐2 vaccines are suggested because headache is a common AE in all vaccines, and it may share nocebo features.

## Acute headache attributed to systemic SARS‐CoV‐2 infection

- Any headache lasting 7–14 days fulfilling criterion C
- Diagnosis of systemic SARS‐CoV‐2 infection
- At least two of the following:
  1. Headache has developed within 14 days after first signs of SARS‐CoV‐2 infection
  2. Either or both of the following:
    - Headache has significantly worsened in parallel with worsening of the systemic SARS‐CoV‐2 infection
    - Headache has significantly improved or resolved in parallel with improvement in or resolution of the systemic SARS‐CoV‐2 infection
- Not better accounted for by another ICHD‐3 diagnosis

### Notes

Headache is diffuse, throbbing, moderate to severe intensity, with bilateral location and resistance to simple analgesics. In a subgroup of people with SARS‐CoV‐2 infection a slight CSF pleocytosis has been observed [28, 95], without other meningitic or encephalitic symptoms or signs. In this case, appropriate exclusion of other causes of CSF cell elevation is required. Headache attributed to first SARS‐CoV‐2 variants usually last 7–14 days, but in newer variants headache may last only a few days [28]. Anosmia and/or ageusia, either total or more often partial, often accompany headache in acute COVID‐19, but not in all cases.

#### Comments

SARS‐CoV‐2 infection may be associated with an increase in migraine attacks, which could indicate the involvement of inflammatory mediators in the pathophysiology of migraine and can impact the intrinsic threshold for upcoming attacks. In this case both migraine and headache attributed to SARS‐CoV‐2 infection should be coded as a dual diagnosis. In case only typical migraine attacks are worsened, the diagnosis remains migraine as primary headache disorder. The same applies for the case of pre‐existing TTH.

Distinguishing acute headache attributed to systemic SARS‐CoV‐2 infection from primary headache disorders such as migraine or TTH might be difficult. Characterization of frequently recurring headache generally requires a headache diary to record information on pain and associated symptoms.

## Chronic headache attributed to systemic SARS‐CoV‐2 infection

Headache lasting for more than 3 months and fulfilling criteria A and B.

- All of the following:

1. Diagnosis of systemic SARS‐CoV‐2 infection
2. Headache has developed within 14 days after the first signs of systemic SARS‐CoV‐2 infection
3. There is no evidence of meningitic or encephalitic involvement

B Not better accounted for by another ICHD‐3 diagnosis

### Notes

In a subgroup of people with SARS‐CoV‐2 infection a slight CSF pleocytosis has been observed [95], without other meningitic or encephalitic symptoms or signs. In this case, appropriate exclusion of other causes of CSF cell elevation is required.

For those people whose headache lasts more than 14 days but <3 months a diagnosis of acute headache attributed to systemic SARS‐CoV‐2 infection should be applied.

### Comments

SARS‐CoV‐2 infection may be associated with an increase in migraine attacks, which could indicate the involvement of inflammatory mediators in the pathophysiology of migraine and can impact the intrinsic threshold for upcoming attacks. In this case both migraine and headache attributed to SARS‐CoV‐2 infection should be coded as a diagnosis. If only typical migraine attacks are worsened, the diagnosis remains migraine as the primary headache disorder. The same applies for the case of pre‐existing TTH.

Distinguishing acute headache attributed to systemic SARS‐CoV‐2 infection from primary headache disorders such as migraine or TTH might be difficult. Characterization of frequently recurring headache generally requires a headache diary to record information on pain and associated symptoms.

## CONFLICT OF INTEREST STATEMENT

The authors declare no conflicts of interest.

## AUTHOR CONTRIBUTIONS

**Dimos Mitsikostas:** Conceptualization; writing – original draft; project administration; supervision; resources; visualization; writing – review and editing; methodology; investigation; validation. **Edoardo Caronna:** Validation; data curation; methodology. **Marina de Tommaso:** Conceptualization; writing – review and editing; formal analysis. **Christina I Deligianni:** Data curation; writing – review and editing. **Esme Ekizoglu:** Data curation; writing – review and editing. **Hayrunnisa Bolay:** Conceptualization; visualization; writing – review and editing. **Carl H. Göbe:** Conceptualization; writing – review and editing; visualization. **Espen Saxhaug Kristoffersen:** Conceptualization; writing – review and editing; visualization. **Christian Lampl:** Conceptualization; visualization; writing – review and editing. **Elena Moro:** Conceptualization; visualization; writing – review and editing. **Patricia Pozo‐Rosich:** Conceptualization; visualization; writing – review and editing. **Johann Sellner:** Conceptualization; writing – review and editing; visualization; validation. **Gisela M. Terwindt:** Conceptualization; writing – review and editing. **Pablo Irimia‐Sieira:** Conceptualization; validation; visualization; writing – review and editing; supervision.

## Supporting information


Appendix S1:

Appendix S2:

Appendix S3:

Appendix S4:

Appendix S5:

## Data Availability

The data that supports the findings of this study are available in the supplementary material of this article
