# Supplementary material for: Headaches and facial pain attributed to SARS‐CoV‐2 infection and vaccination: a systematic review
Source: Eur J Neurol. 2024 Feb 28;31(6):e16251. doi: 10.1111/ene.16251 (PMC11235838; doi:10.1111/ene.16251)
Supplement: Supplementary file 1 — Appendix S1: [file ENE-31-e16251-s005.docx]

**Appendix No1**

**List of References for PICO 1**

1. Wanga V, Chevinsky JR, Dimitrov LV, et al Long-Term Symptoms Among Adults Tested for SARS-CoV-2 — United States, January 2020–April 2021. MMWR Morb Mortal Wkly Rep. 2021; 70(36):1235-1241.
2. Hossain MA, Hossain KMA, Saunders K, et al. Prevalence of Long COVID symptoms in Bangladesh: a prospective Inception Cohort Study of COVID-19 survivors. BMJ Glob Health. 2021;6(12):e006838.
3. Wohl DA, Barzin AH, Napravnik S,et al. COVID-19 symptoms at time of testing and association with positivity among outpatients tested for SARS-CoV-2. PLoS One. 2021;16(12):e0260879.
4. Sampaio Rocha-Filho PA, Albuquerque PM, Carvalho LCLS, Dandara Pereira Gama M, Magalhães JE. Headache, anosmia, ageusia and other neurological symptoms in COVID-19: a cross-sectional study. J Headache Pain. 2022;23(1):2.
5. Hussein M, Fathy W, Eid RA, et al. Relative Frequency and Risk Factors of COVID-19 Related Headache in a Sample of Egyptian Population: A Hospital-Based Study. Pain Med. 2021;22(9):2092-2099
6. Filho FFD, Chaves EBM, D'Avila KG, Neyeloff JL, Dos Santos RP, Silva DR. Clinical characteristics and outcomes of healthcare workers with COVID-19 pre- and postvaccination. J Med Virol. 2022;94(11):5279-5283.
7. Kleineberg NN, Knauss S, Gülke E, et al. Neurological symptoms and complications in predominantly hospitalized COVID-19 patients: Results of the European multinational Lean European Open Survey on SARS-Infected Patients (LEOSS). Eur J Neurol. 2021;28(12):3925-3937.
8. Abdelrahman MM, Abd-Elrahman NM, Bakheet TM. Persistence of symptoms after improvement of acute COVID19 infection, a longitudinal study. J Med Virol. 2021;93(10):5942-5946.
9. Nielsen KJ, Vestergaard JM, Schlünssen V, et al. Day-by-day symptoms following positive and negative PCR tests for SARS-CoV-2 in non-hospitalized healthcare workers: A 90-day follow-up study. Int J Infect Dis. 2021;108:382-390.
10. Fernández-de-Las-Peñas C, Cuadrado ML, Gómez-Mayordomo V, et al. Headache as a COVID-19 onset symptom and post-COVID-19 symptom in hospitalized COVID-19 survivors infected with the Wuhan, Alpha, or Delta SARS-CoV-2 variants. Headache. 2022;62(9):1148-1152.
11. Wu Q, Ailshire JA, Crimmins EM. Long COVID and symptom trajectory in a representative sample of Americans in the first year of the pandemic. Sci Rep. 2022;12(1):11647.
12. Bhandari S, Rankawat G, Diwakar M, Gupta V. Extra-pulmonary Manifestations of COVID-19 in Western India. J Assoc Physicians India. 2022;70(3):11-12
13. Sebo P, Tudrej B, Lourdaux J, et al. Cross sectional study of the clinical characteristics of French primary care patients with COVID-19. Sci Rep. 2021;11(1):12492.
14. Sandmann FG, Tessier E, Lacy J, et al. Long-Term Health-Related Quality of Life in Non-Hospitalized Coronavirus Disease 2019 (COVID-19) Cases With Confirmed Severe Acute Respiratory Syndrome Coronavirus 2 (SARS-CoV-2) Infection in England: Longitudinal Analysis and Cross-Sectional Comparison With Controls. Clin Infect Dis. 2022;75(1):e962-e973.
15. Zulu JE, Banda D, Hines JZ, et al. Two-month follow-up of persons with SARS-CoV-2 infection-Zambia, September 2020: a cohort study. Pan Afr Med J. 2022;41:26.
16. Kirca F, Aydoğan S, Gözalan A, et al. Comparison of clinical characteristics of wild-type SARS-CoV-2 and Omicron. Rev Assoc Med Bras (1992). 2022;68(10):1476-1480.
17. Tiwari B, Ghimire M, Bhatta G, et al. Persistent Symptoms in Non-critical COVID-19 Patients at Two Months Follow-Up in a District Hospital: A Descriptive Cross-sectional Study. JNMA J Nepal Med Assoc. 2021;59(238):550-553.
18. de Miranda DAP, Gomes SVC, Filgueiras PS, et al. Long COVID-19 syndrome: a 14-months longitudinal study during the two first epidemic peaks in Southeast Brazil. Trans R Soc Trop Med Hyg. 2022;116(11):1007-1014.
19. Karadaş Ö, Öztürk B, Sonkaya AR. A prospective clinical study of detailed neurological manifestations in patients with COVID-19. Neurol Sci. 2020;41(8):1991-1995.
20. Silverberg JI, Zyskind I, Naiditch H, et al. Predictors of chronic COVID-19 symptoms in a community-based cohort of adults. PLoS One. 2022;17(8):e0271310.
21. García-Azorín D, Abildúa MJA, Aguirre MEE, et al. Neurological presentations of COVID-19: Findings from the Spanish Society of Neurology neuroCOVID-19 registry. J Neurol Sci. 2021;423:117283.
22. Leal FE, Mendes-Correa MC, Buss LF, et al. Clinical features and natural history of the first 2073 suspected COVID-19 cases in the Corona São Caetano primary care programme: a prospective cohort study. BMJ Open. 2021;11(1):e042745.
23. Marcolino MS, Anschau F, Kopittke L, et al. Frequency and burden of neurological manifestations upon hospital presentation in COVID-19 patients: Findings from a large Brazilian cohort. J Neurol Sci. 2022;443:120485.
24. Ancochea J, Izquierdo JL, Soriano JB. Evidence of Gender Differences in the Diagnosis and Management of Coronavirus Disease 2019 Patients: An Analysis of Electronic Health Records Using Natural Language Processing and Machine Learning. J Womens Health (Larchmt). 2021;30(3):393-404.
25. Nehme M, Braillard O, Chappuis F, et al. One‐year persistent symptoms and functional impairment in SARS‐CoV‐2 positive and negative individuals. J Intern Med. 2022;292(1):103-115.
26. Schönfeld D, Arias S, Bossio JC, Fernández H, Gozal D, Pérez-Chada D. Clinical presentation and outcomes of the first patients with COVID-19 in Argentina: Results of 207079 cases from a national database. PLoS One. 2021;16(2):e0246793.
27. Fernández-de-Las-Peñas C, Pellicer-Valero OJ, Navarro-Pardo E, et al. Symptoms Experienced at the Acute Phase of SARS-CoV-2 Infection as Risk Factor of Long-term Post-COVID Symptoms: The LONG-COVID-EXP-CM Multicenter Study. Int J Infect Dis. 2022;116:241-244.
28. Viera-Segura O, Vega-Magaña N, García-Chagollán M, et al. A Comprehensive Descriptive Epidemiological and Clinical Analysis of SARS-CoV-2 in West-Mexico during COVID-19 Pandemic 2020
29. Kläser K, Molteni E, Graham M, et al. COVID-19 due to the B.1.617.2 (Delta) variant compared to B.1.1.7 (Alpha) variant of SARS-CoV-2: a prospective observational cohort study. Sci Rep. 2022;12(1):10904.
30. Khan SM, Farland LV, Catalfamo CJ, et al. Elucidating symptoms of COVID-19 illness in the Arizona CoVHORT: a longitudinal cohort study. BMJ Open. 2022;12(1):e053403
31. Oliveira V, Seabra M, Rodrigues R, et al. Neuro‐COVID frequency and short‐term outcome in the Northern Portuguese population. Eur J Neurol. 2021;28(10):3360-3368
32. Ziauddeen N, Gurdasani D, O'Hara ME, et al. Characteristics and impact of Long Covid: Findings from an online survey. PLoS One. 2022;17(3):e0264331.
33. Dryden M, Mudara C, Vika C, et al. Post-COVID-19 condition 3 months after hospitalisation with SARS-CoV-2 in South Africa: a prospective cohort study. Lancet Glob Health. 2022;10(9):e1247-e1256.
34. Simian D, Martínez M, Dreyse J, Chomali M, Retamal M, Labarca G. Clinical characteristics and predictors of hospitalization among 7,108 ambulatory patients with positive RT-PCR for SARS-CoV-2 during the acute pandemic period. J Bras Pneumol. 2021;47(4):e20210131.
35. Chudzik M, Babicki M, Kapusta J, et al. Long-COVID Clinical Features and Risk Factors: A Retrospective Analysis of Patients from the STOP-COVID Registry of the PoLoCOV Study. Viruses. 2022;14(8):1755.
36. Romero-Sánchez CM, Díaz-Maroto I, Fernández-Díaz E, et al. Neurologic manifestations in hospitalized patients with COVID-19: The ALBACOVID registry. Neurology. 2020;95(8):e1060-e1070.
37. Sampaio Rocha-Filho PA, Magalhães JE, Fernandes Silva D, et al. Neurological manifestations as prognostic factors in COVID-19: a retrospective cohort study. Acta Neurol Belg. 2022;122(3):725-733.
38. Lechien JR, Chiesa-Estomba CM, Place S, et al. Clinical and epidemiological characteristics of 1420 European patients with mild‐to‐moderate coronavirus disease 2019. J Intern Med. 2020;288(3):335-344.
39. Tian S, Hu N, Lou J, et al. Characteristics of COVID-19 infection in Beijing. J Infect. 2020;80(4):401-406.
40. Leulseged TW, Abebe KG, Hassen IS, et al. COVID-19 disease severity and associated factors among Ethiopian patients: A study of the millennium COVID-19 care center. PLoS One. 2022;17(1):e0262896.
41. Schäfer E, Scheer C, Saljé K, et al. Course of disease and risk factors for hospitalization in outpatients with a SARS-CoV-2 infection. Sci Rep. 2022;12(1):7249.
42. Romero-Duarte Á, Rivera-Izquierdo M, Guerrero-Fernández de Alba I, et al. Sequelae, persistent symptomatology and outcomes after COVID-19 hospitalization: the ANCOHVID multicentre 6-month follow-up study. BMC Med. 2021;19(1):129.
43. Graham EL, Clark JR, Orban ZS, et al. Persistent neurologic symptoms and cognitive dysfunction in non‐hospitalized Covid‐19 “long haulers”. Ann Clin Transl Neurol. 2021;8(5):1073-1085.
44. Chou SH, Beghi E, Helbok R, et al. Global Incidence of Neurological Manifestations Among Patients Hospitalized With COVID-19-A Report for the GCS-NeuroCOVID Consortium and the ENERGY Consortium. JAMA Netw Open. 2021;4(5):e2112131.
45. Taquet M, Dercon Q, Luciano S, Geddes JR, Husain M, Harrison PJ. Incidence, co-occurrence, and evolution of long-COVID features: A 6-month retrospective cohort study of 273,618 survivors of COVID-19. PLoS Med. 2021;18(9):e1003773.
46. Nejad JH, Allahyari F, Hosseinzadeh R, Heiat M, Ranjbar R. Neurological symptoms of COVID-19 infection; a cross-sectional study on hospitalized COVID-19 patients in Iran. Clin Neurol Neurosurg. 2021;210:106985.
47. Eythorsson E, Helgason D, Ingvarsson RF, et al. Clinical spectrum of coronavirus disease 2019 in Iceland: population based cohort study. BMJ. 2020;371:m4529.
48. Köseoğlu Toksoy C, Yavuz T, Orhan S, et al. Neurological symptoms and findings in COVID-19: a prospective clinical study. Neurol Res. 2022;44(1):1-6.
49. Mizrahi B, Shilo S, Rossman H, et al. Longitudinal symptom dynamics of COVID-19 infection. Nat Commun. 2020;11(1):6208.
50. Knox N, Lee CS, Moon JY, Cohen SP. Pain Manifestations of COVID-19 and Their Association With Mortality: A Multicenter Prospective Observational Study. Mayo Clin Proc. 2021;96(4):943-951.
51. García-Azorín D, Trigo J, Martínez-Pías E, et al. Neurological symptoms in Covid-19 patients in the emergency department. Brain Behav. 2021;11(4):e02058.
52. Barbara JM, Gatt J, Xuereb RA,et al. Clinical outcomes at medium-term follow-up of COVID-19. J R Coll Physicians Edinb. 2022;52(3):220-227.
53. Adham D, Habibzadeh S, Ghobadi H, Jajin SA, Abbasi-Ghahramanloo A, Moradi-Asl E. Epidemiological characteristics and mortality risk factors among COVID-19 patients in Ardabil, Northwest of Iran. BMC Emerg Med. 2021;21(1):67.
54. Perez Duque M, Saad NJ, Lucaccioni H, et al. Clinical and hospitalisation predictors of COVID-19 in the first month of the pandemic, Portugal. PLoS One. 2021;16(11):e0260249.
55. Lhendup K, Tsheten T, Rinzin T, Wangdi K. Risk Factors of Symptomatic COVID-19 in Samtse District, Bhutan. Front Public Health. 2022;10:857084.
56. Tabacof L, Tosto-Mancuso J, Wood J, et al. Post-acute COVID-19 Syndrome Negatively Impacts Physical Function, Cognitive Function, Health-Related Quality of Life, and Participation. Am J Phys Med Rehabil. 2022;101(1):48-52.
57. O'Keefe JB, Tong EJ, O'Keefe GD, Tong DC. Description of symptom course in a telemedicine monitoring clinic for acute symptomatic COVID-19: a retrospective cohort study. BMJ Open. 2021;11(3):e044154.
58. Pullen MF, Skipper CP, Hullsiek KH, et al. Symptoms of COVID-19 Outpatients in the United States. Open Forum Infect Dis. 2020;7(7):ofaa271.
59. Kuodi P, Gorelik Y, Zayyad H, et al. Association between BNT162b2 vaccination and reported incidence of post-COVID-19 symptoms: cross-sectional study 2020-21, Israel. NPJ Vaccines. 2022;7(1):101.
60. Zuschlag D, Grandt D, Custodis F, Braun C, Häuser W. Spontaneously reported persistent symptoms related to coronavirus disease 2019 one year after hospital discharge. Schmerz. 2022;36(5):315-325.
61. Blair PW, Brown DM, Jang M, et al. The Clinical Course of COVID-19 in the Outpatient Setting: A Prospective Cohort Study. Open Forum Infect Dis. 2021;8(2):ofab007.
62. Titze-de-Almeida R, da Cunha TR, Dos Santos Silva LD, et al. Persistent, new-onset symptoms and mental health complaints in Long COVID in a Brazilian cohort of non-hospitalized patients. BMC Infect Dis. 2022;22(1):133.
63. Trigo J, García-Azorín D, Planchuelo-Gómez Á, et al. Factors associated with the presence of headache in hospitalized COVID-19 patients and impact on prognosis: a retrospective cohort study. J Headache Pain. 2020;21(1):94.
64. Vihta KD, Pouwels KB, Peto TEA, et al. Symptoms and Severe Acute Respiratory Syndrome Coronavirus 2 (SARS-CoV-2) Positivity in the General Population in the United Kingdom. Clin Infect Dis. 2022;75(1):e329-e337.
65. Alkeridy WA, Alanazy MH, Alamri N, Alqahtani A, Alhazzani A, Muayqil T. The Common Neurological Presentations and Clinical Outcomes of Coronavirus Disease 2019 in Saudi Arabia. Front Neurol. 2021;12:737328.
66. Zhang J, Yang S, Xu Y, et al.Epidemiological and clinical characteristics of imported cases of COVID-19: a multicenter study. BMC Infect Dis. 2021;21(1):406
67. Ortiz-Prado E, Simbaña-Rivera K, Barreno LG, et al. Epidemiological, socio-demographic and clinical features of the early phase of the COVID-19 epidemic in Ecuador. PLoS Negl Trop Dis. 2021;15(1):e0008958.
68. Wiegele PN, Kabar I, Kerschke L, et al. Symptom Diary–Based Analysis of Disease Course among Patients with Mild Coronavirus Disease, Germany, 2020. Emerg Infect Dis. 2021;27(5):1353-1361.
69. Kim HK, Cho YJ, Lee SY. Neurological Manifestations in Patients with COVID-19: Experiences from the Central Infectious Diseases Hospital in South Korea. J Clin Neurol. 2021;17(3):435-442.
70. García-Moncó JC, Cabrera Muras A, Erburu Iriarte M, et al. Neurologic Manifestations in a Prospective Unselected Series of Hospitalized Patients With COVID-19. Neurol Clin Pract. 2021;11(2):e64-e72.
71. Rokni M, Ahmadikia K, Asghari S, Mashaei S, Hassanali F. Comparison of clinical, para-clinical and laboratory findings in survived and deceased patients with COVID-19: diagnostic role of inflammatory indications in determining the severity of illness. BMC Infect Dis. 2020;20(1):869.
72. Vahey GM, Marshall KE, McDonald E, et al. Symptom Profiles and Progression in Hospitalized and Nonhospitalized Patients with Coronavirus Disease, Colorado, USA, 2020. Emerg Infect Dis. 2021;27(2):385-395.
73. Sisay G, Mantefardo B, Beyene A. Time from symptom onset to severe COVID-19 and risk factors among patients in Southern Ethiopia: a survival analysis. J Int Med Res. 2022;50(8):3000605221119366.
74. Yan N, Xu Z, Mei B, Gao Y, Lv D, Zhang J. Neurological Implications of Non-critically Ill Patients With Coronavirus Disease 2019 in a Fangcang Shelter Hospital in Wuhan, China. Front Neurol. 2020;11:895.
75. Amanat M, Rezaei N, Roozbeh M, et al. Neurological manifestations as the predictors of severity and mortality in hospitalized individuals with COVID-19: a multicenter prospective clinical study
76. Di Stadio A, Brenner MJ, De Luca P, et al. Olfactory Dysfunction, Headache, and Mental Clouding in Adults with Long-COVID-19: What Is the Link between Cognition and Olfaction? A Cross-Sectional Study. Brain Sci. 2022;12(2):154.
77. Nalleballe K, Reddy Onteddu S, Sharma R, et al. Spectrum of neuropsychiatric manifestations in COVID-19. Brain Behav Immun. 2020;88:71-74
78. Oguz-Akarsu E, Gullu G, Kilic E, et al. Insight into pain syndromes in acute phase of mild‐to‐moderate COVID‐19: Frequency, clinical characteristics, and associated factors. Eur J Pain. 2022;26(2):492-504.
79. Zhang X, Cai H, Hu J, et al. Epidemiological, clinical characteristics of cases of SARS-CoV-2 infection with abnormal imaging findings
80. Vahabizad F, Togha M, Ariyanfar S, et al. Clinical characteristics of 365 hospitalized COVID-19 patients with neurological symptoms: an observational study. Acta Neurol Belg. 2022;1-7.
81. Saad NJ, Moek F, Steitz F, et al. A longitudinal study on symptom duration and 60-day clinical course in non-hospitalised COVID-19 cases in Berlin, Germany, March to May, 2020. Euro Surveill. 2021;26(43):2001757.
82. Garg R, Jain R, Sodani A, et al. Neurological Symptoms as Initial Manifestation of Covid-19 – An Observational Study. Ann Indian Acad Neurol. 2020;23(4):482-486.
83. Gogu AE, Motoc AG, Stroe AZ, et al. Clinical Spectrum and Neuroimagistic Features in Hospitalized Patients with Neurological Disorders and Concomitant Coronavirus-19 Infection. Brain Sci. 2021;11(9):1138.
84. Wnuk M, Sawczyńska K, Kęsek T, et al. Neurological symptoms in hospitalised patients with COVID-19 and their association with in-hospital mortality. Neurol Neurochir Pol. 2021;55(3):314-321.
85. Howe HL, Rankin DA, Bloos SM, et al. Persistence of Long COVID in SARS-CoV-2 Confirmed Cases One-Year Post Infection. Open Forum Infect Dis. 2021;8:S252–S253.
86. Lapostolle F, Schneider E, Vianu I, et al. Clinical features of 1487 COVID-19 patients with outpatient management in the Greater Paris: the COVID-call study. Intern Emerg Med. 2020;15(5):813-817.
87. Gutiérrez-Canales LG, Muñoz-Corona C, Barrera-Chávez I, Viloria-Álvarez C, Macías AE, Guaní-Guerra E. Quality of Life and Persistence of Symptoms in Outpatients after Recovery from COVID-19. Medicina (Kaunas). 2022;58(12):1795.
88. Takakura K, Suka M, Kajihara M, Koido S. Clinical features, therapeutic outcomes, and recovery period of long COVID. J Med Virol. 2023;95(1):e28316.
89. Kumar J, Makheja K, Rahul F, et al. Long-Term Neurological Impact of COVID-19. Cureus. 2021;13(9):e18131.
90. Ali L, Muhammad A, Khan A, et al. Acute Neurological Manifestations of COVID-19 Patients From Three Tertiary Care Hospitals in Qatar. Cureus. 2022;14(3):e23150.
91. Cyrille TM, Serge SM, Brice TMJ, et al. Clinical presentation of COVID-19 at the time of testing and factors associated with pre-symptomatic cases in Cameroon. IJID Reg. 2022;4:33-41.
92. Khan TS, Shirin T, Alam AN, Shakhider MAH, Haque F. Follow-up of practiced treatment regimens and health conditions of patients following recovery from COVID-19 residing in Dhaka City: a survey-based, descriptive, cross-sectional study. IJID Reg. 2022;3:68-75.
93. Johnson BJ, Salonen B, O'Byrne TJ, et al. Patient factors associated with COVID‐19 loss of taste or smell patient factors in smell/taste loss COVID‐19. Laryngoscope Investig Otolaryngol. 2022;7(6):1688-1694.
94. Bin Abdulrahman KA, Bamosa AO, Aseri KS, Bukhari AI, Masuadi EM. Clinical Presentation of Asymptomatic and Mild SARS-CoV-2 Infection in Riyadh, Saudi Arabia. J Multidiscip Healthc. 2021;14:1341-1347.
95. Martinez F, Muñoz S, Guerrero-Nancuante C, Taramasco C. Sensitivity and Specificity of Patient-Reported Clinical Manifestations to Diagnose COVID-19 in Adults from a National Database in Chile: A Cross-Sectional Study. Biology (Basel). 2022;11(8):1136.
96. González-Andrade F. Post-COVID-19 conditions in Ecuadorian patients: an observational study. Lancet Reg Health Am. 2022;5:100088.
97. Knight D, Downes K, Munipalli B, et al. Symptoms and Clinical Outcomes of Coronavirus Disease 2019 in the Outpatient Setting. SN Compr Clin Med. 2021;3(1):247-254.
98. Alkwai HM, Khalifa AM, Ahmed AM, et al. Persistence of COVID-19 symptoms beyond 3 months and the delayed return to the usual state of health in Saudi Arabia: A cross-sectional study. SAGE Open Med. 2022;10:20503121221129918.
99. Mahmoud N, Radwan N, Alkattan A, et al. Post-COVID-19 syndrome: nature of symptoms and associated factors. Z Gesundh Wiss. 2023;1-6.
100. Patel JR, Amick BC, Vyas KS, Bircan E, Boothe D, Nembhard WN. Racial disparities in symptomatology and outcomes of COVID-19 among adults of Arkansas. Prev Med Rep. 2022;28:101840.
101. Zayet S, Zahra H, Royer PY, et al. Post-COVID-19 Syndrome: Nine Months after SARS-CoV-2 Infection in a Cohort of 354 Patients: Data from the First Wave of COVID-19 in Nord Franche-Comté Hospital, France. Microorganisms. 2021;9(8):1719.
102. Akıncı T. Post-discharge persistent headache and smell or taste dysfunction after hospitalisation for COVID-19: a single-centre study. Ir J Med Sci. 2023;192(1):369-375.
103. Sansone D, Tassinari A, Valentinotti R, et al. Persistence of Symptoms 15 Months since COVID-19 Diagnosis: Prevalence, Risk Factors and Residual Work Ability. Life (Basel). 2022;13(1):97.
104. Chudzik M, Lewek J, Kapusta J, Banach M, Jankowski P, Bielecka-Dabrowa A. Predictors of Long COVID in Patients without Comorbidities: Data from the Polish Long-COVID Cardiovascular (PoLoCOV-CVD) Study. J Clin Med. 2022;11(17):4980
105. Senjam SS, Balhara YPS, Kumar P, et al. A Comprehensive Assessment of Self-Reported Post COVID-19 Symptoms Among Beneficiaries of Hospital Employee Scheme at a Tertiary Healthcare Institution in Northern India. Int J Gen Med. 2022;15:7355-7372.
106. Yelin D, Margalit I, Nehme M, et al. Patterns of Long COVID Symptoms: A Multi-Center Cross Sectional Study. J Clin Med. 2022;11(4):898.
107. Domènech-Montoliu S, Puig-Barberà J, Pac-Sa MR, et al. Complications Post-COVID-19 and Risk Factors among Patients after Six Months of a SARS-CoV-2 Infection: A Population-Based Prospective Cohort Study. Epidemiologia (Basel). 2022;3(1):49-67.
108. Kim J, Heo N, Kang H. Sex-Based Differences in Outcomes of Coronavirus Disease 2019 (COVID-19) in Korea. Asian Nurs Res (Korean Soc Nurs Sci). 2022;16(4):224-30.
109. Merza MA, Aswad SM, Sulaiman HM, Abdulah DM, Rasheed WS, Taib NI. Clinical and epidemiological characteristics and outcomes of Coronavirus disease-19 patients in a large longitudinal study. Int J Health Sci (Qassim). 2021;15(4):29-41.
110. García S, Cuatepotzo-Burgos FM, Toledo-Lozano CG, et al. Neurological Manifestations and Outcomes in a Retrospective Cohort of Mexican Inpatients with SARS-CoV-2 Pneumonia: Design of a Risk Profile. Healthcare (Basel). 2021;9(11):1501.
111. Otsuka Y, Tokumasu K, Nakano Y, et al. Clinical Characteristics of Japanese Patients Who Visited a COVID-19 Aftercare Clinic for Post-Acute Sequelae of COVID-19/Long COVID. Cureus. 2021;13(10):e18568.
112. Carcamo Garcia MH, Garcia Choza DD, Salazar Linares BJ, Diaz MM. Neurological manifestations of patients with mild-to-moderate COVID-19 attending a public hospital in Lima, Peru. eNeurologicalSci. 2021;23:100338.
113. Vaish A, Ray S, Tyson B. A Study on the Correlations Between Comorbid Disease Conditions and Central and Peripheral Neurological Manifestations of COVID-19. Cureus. 2022;14(10):e29838.
114. Rodríguez-Pérez MP, Sánchez-Herrera-Baeza P, Rodríguez-Ledo P, Serrada-Tejeda S, García-Bravo C, Pérez-de-Heredia-Torres M. Headaches and Dizziness as Disabling, Persistent Symptoms in Patients with Long COVID–A National Multicentre Study. J Clin Med. 2022;11(19):5904.
115. Pereira DN, Bicalho MAC, Jorge AO, et al. Neurological manifestations by sex and age group in COVID-19 inhospital patients. eNeurologicalSci. 2022;28:100419.
116. Al-Hussain OH. Complications and Comorbidities in COVID-19 Patients: A Comparative study. Cureus. 2022;14(8):e28614.
117. Ashrafi F, Ommi D, Zali A, et al. Neurological Manifestations and their Correlated Factors in COVID-19 Patients; a Cross-Sectional Study. Arch Acad Emerg Med. 2021;9(1):e34.
118. Marra DE, Busl KM, Robinson CP, et al. Examination of Early CNS Symptoms and Severe Coronavirus Disease 2019: A Multicenter Observational Case Series. Crit Care Explor. 2021;3(6):e0456.
119. Anjana NKN, Annie TT, Siba S, Meenu MS, Chintha S, Anish TSN. Manifestations and risk factors of post COVID syndrome among COVID-19 patients presented with minimal symptoms – A study from Kerala, India. J Family Med Prim Care. 2021;10(11):4023-4029.
120. Orrù G, Bertelloni D, Diolaiuti F, et al. Long-COVID Syndrome? A Study on the Persistence of Neurological, Psychological and Physiological Symptoms. Healthcare (Basel). 2021;9(5):575.
121. Rocha-Filho PAS, Magalhães JE. Headache associated with COVID-19: Frequency, characteristics and association with anosmia and ageusia. Cephalalgia. 2020;40(13):1443-1451.
122. Blair JE, Gotimukul A, Wang F, et al. Mild to moderate COVID-19 illness in adult outpatients: Characteristics, symptoms, and outcomes in the first 4 weeks of illness. Medicine (Baltimore). 2021;100(24):e26371.
123. García-Azorín D, Sierra Á, Trigo J, et al. Frequency and phenotype of headache in covid-19: a study of 2194 patients. Sci Rep. 2021;11(1):14674.
124. Gonzalez-Martinez A, Fanjul V, Ramos C, et al. Headache during SARS-CoV-2 infection as an early symptom associated with a more benign course of disease: a case-control study. Eur J Neurol. 2021;28(10):3426-3436.
125. Mutiawati E, Kusuma HI, Fahriani M, Harapan H, Syahrul S, Musadir N. Headache in Post-COVID-19 Patients: Its Characteristics and Relationship with the Quality of Life. Medicina (Kaunas). 2022;58(10):1500.
126. Caronna E, Ballvé A, Llauradó A, et al. Headache: A striking prodromal and persistent symptom, predictive of COVID-19 clinical evolution. Cephalalgia. 2020;40(13):1410-1421.
127. Karadaş Ö, Öztürk B, Sonkaya AR, Taşdelen B, Özge A, Bolay H. Latent class cluster analysis identified hidden headache phenotypes in COVID-19: impact of pulmonary infiltration and IL-6. Neurol Sci. 2021;42(5):1665-1673.
128. Sharma M, Menon B. Headache Incidence and Characteristics in COVID-19 Patients: A Hospital-Based Study. Ann Indian Acad Neurol. 2022;25(1):88-91.
129. Membrilla JA, de Lorenzo Í, Sastre M, Díaz de Terán J. Headache as a Cardinal Symptom of Coronavirus Disease 2019: A  Cross-Sectional Study. Headache. 2020;60(10):2176-2191.
130. Poncet-Megemont L, Paris P, Tronchere A, et al. High Prevalence of Headaches During Covid-19 Infection: A Retrospective  Cohort Study. Headache. 2020;60(10):2578-2582.
131. Kacem I, Gharbi A, Harizi C, et al. Characteristics, onset, and evolution of neurological symptoms in patients with COVID-19. Neurol Sci. 2021;42(1):39-46.
132. Souza DD, Shivde S, Awatare P, et al. Headaches associated with acute SARS-CoV-2 infection: A prospective cross-sectional study. SAGE Open Med. 2021;9:20503121211050227.
133. Karyakarte RP, Das R, Taji N, et al. An Early and Preliminary Assessment of the Clinical Severity of the Emerging SARS-CoV-2 Omicron Variants in Maharashtra, India. Cureus. 2022;14(11):e31352.
134. Garcia-Azorin D, Layos-Romero A, Porta-Etessam J, et al. Post-COVID-19 persistent headache: A multicentric 9-months follow-up study of 905 patients. Cephalalgia. 2022;42(8):804-809.
135. Vacchiano V, Riguzzi P, Volpi L, et al. Early neurological manifestations of hospitalized COVID-19 patients. Neurol Sci. 2020;41(8):2029-2031.
136. Kacprzak A, Malczewski D, Domitrz I. Headache Attributed to SARS-CoV-2 Infection or COVID-19 Related Headache-Not Migraine-like Problem-Original Research. Brain Sci. 2021;11(11):1406.
137. Straburzyński M, Nowaczewska M, Budrewicz S, Waliszewska-Prosół M. COVID-19-related headache and sinonasal inflammation: A longitudinal study analysing the role of acute rhinosinusitis and ICHD-3 classification difficulties in SARS-CoV-2 infection. Cephalalgia. 2022;42(3):218-228.
138. Sahin BE, Celikbilek A, Kocak Y, Hizmali L. Patterns of COVID-19-related headache: A cross-sectional study. Clin Neurol Neurosurg. 2022;219:107339.
