# Supplementary material for: Headaches and facial pain attributed to SARS‐CoV‐2 infection and vaccination: a systematic review
Source: Eur J Neurol. 2024 Feb 28;31(6):e16251. doi: 10.1111/ene.16251 (PMC11235838; doi:10.1111/ene.16251)
Supplement: Supplementary file 2 — Appendix S2: [file ENE-31-e16251-s003.docx]

**Appendix No2**

**List of References for PICO 2**

1. Caronna E, Ballvé A, Llauradó A, et al. Headache: A striking prodromal and persistent symptom, predictive of COVID-19 clinical evolution. Cephalalgia. 2020;40(13):1410-1421.
2. Rocha-Filho PAS, Magalhães JE. Headache associated with COVID-19: Frequency, characteristics, and association with anosmia and ageusia. Cephalalgia. 2020;40(13):1443-1451.
3. García-Azorín D, Sierra Á, Trigo J, et al. Frequency and phenotype of headache in covid-19: a study of 2194 patients. Sci Rep. 2021;11(1):14674.
4. Gonzalez-Martinez A, Fanjul V, Ramos C, et al. Headache during SARS-CoV-2 infection as an early symptom associated with a more benign course of disease: a case-control study. Eur J Neurol. 2021;28(10):3426-3436.
5. Magdy R, Elmazny A, Soliman SH, Elsebaie EH, Ali SH, Abdel Fattah AM, et al. Post-COVID-19 neuropsychiatric manifestations among COVID-19 survivors suffering from migraine: a case–control study. J Headache Pain. 2022;23(1):101.
6. Blair JE, Gotimukul A, Wang F, et al. Mild to moderate COVID-19 illness in adult outpatients: Characteristics, symptoms, and outcomes in the first 4 weeks of illness. Medicine (Baltimore). 2021;100(24):e26371.
7. Mutiawati E, Kusuma HI, Fahriani M, Harapan H, Syahrul S, Musadir N. Headache in Post-COVID-19 Patients: Its Characteristics and Relationship with the Quality of Life. Medicina (Kaunas). 2022;58(10):1500.
8. Karadaş Ö, Öztürk B, Sonkaya AR, Taşdelen B, Özge A, Bolay H. Latent class cluster analysis identified hidden headache phenotypes in COVID-19: impact of pulmonary infiltration and IL-6. Neurol Sci. 2021;42(5):1665-1673.
9. Sharma M, Menon B. Headache Incidence and Characteristics in COVID-19 Patients: A Hospital-Based Study. Ann Indian Acad Neurol. 2022;25(1):88-91.
10. Membrilla JA, de Lorenzo Í, Sastre M, Díaz de Terán J. Headache as a Cardinal Symptom of Coronavirus Disease 2019: A  Cross-Sectional Study. Headache. 2020;60(10):2176-2191.
11. Poncet-Megemont L, Paris P, Tronchere A, et al. High Prevalence of Headaches During Covid-19 Infection: A Retrospective  Cohort Study. Headache. 2020;60(10):2578-2582.
12. Kacem I, Gharbi A, Harizi C, et al. Characteristics, onset, and evolution of neurological symptoms in patients with COVID-19. Neurol Sci. 2021;42(1):39-46.
13. Fernández-de-Las-Peñas C, Gómez-Mayordomo V, Cuadrado ML, et al. The presence of headache at onset in SARS-CoV-2 infection is associated with long-term post-COVID headache and fatigue: A case-control study. Cephalalgia. 2021;41(13):1332-1341.
14. Souza DD, Shivde S, Awatare P, et al. Headaches associated with acute SARS-CoV-2 infection: A prospective cross-sectional study. SAGE Open Med. 2021;9:20503121211050227.
15. Karyakarte RP, Das R, Taji N, et al. An Early and Preliminary Assessment of the Clinical Severity of the Emerging SARS-CoV-2 Omicron Variants in Maharashtra, India. Cureus. 2022;14(11):e31352.
16. Garcia-Azorin D, Layos-Romero A, Porta-Etessam J, et al. Post-COVID-19 persistent headache: A multicentric 9-months follow-up study of 905 patients. Cephalalgia. 2022;42(8):804-809.
17. Vacchiano V, Riguzzi P, Volpi L, et al. Early neurological manifestations of hospitalized COVID-19 patients. Neurol Sci. 2020;41(8):2029-2031.
18. Kacprzak A, Malczewski D, Domitrz I. Headache Attributed to SARS-CoV-2 Infection or COVID-19 Related Headache-Not Migraine-like Problem-Original Research. Brain Sci. 2021;11(11):1406.
19. Straburzyński M, Nowaczewska M, Budrewicz S, Waliszewska-Prosół M. COVID-19-related headache and sinonasal inflammation: A longitudinal study analysing the role of acute rhinosinusitis and ICHD-3 classification difficulties in SARS-CoV-2 infection. Cephalalgia. 2022;42(3):218-228.
20. Sahin BE, Celikbilek A, Kocak Y, Hizmali L. Patterns of COVID-19-related headache: A cross-sectional study. Clin Neurol Neurosurg. 2022;219:107339.
21. Dos Anjos de Paula RC, de Maria Frota Vasconcelos T, da Costa FBS, et al. Characterization of Headache in COVID-19: a Retrospective Multicenter Study. Mol Neurobiol. 2021;58(9):4487-4494.
22. López JT, García-Azorín D, Planchuelo-Gómez Á, García-Iglesias C, Dueñas-Gutiérrez C, Guerrero ÁL. Phenotypic characterization of acute headache attributed to SARS-CoV-2: An ICHD-3 validation study on 106 hospitalized patients. Cephalalgia. 2020;40(13):1432–42.
23. Moskatel LS, Smirnoff L. Protracted headache after COVID-19: A case series of 31 patients from a tertiary headache center. Headache. 2022;62(7):903-907.
24. García-Azorín D, Trigo J, Talavera B, et al. Frequency and Type of Red Flags in Patients With Covid-19 and Headache: A Series of 104 Hospitalized Patients. Headache. 2020; 60(8):1664-1672
25. Uygun Ö, Ertaş M, Ekizoğlu E, et al. Headache characteristics in COVID-19 pandemic-a survey study. J Headache Pain. 2020;21(1):121.
26. Bolay H, Karadas Ö, Oztürk B, et al. HMGB1, NLRP3, IL-6 and ACE2 levels are elevated in COVID-19 with headache: a window to the infection-related headache mechanism. J Headache Pain. 2021;22(1):94.
27. Fernández-de-Las-Peñas C, Gómez-Mayordomo V, García-Azorín D, et al. Previous History of Migraine Is Associated With Fatigue, but Not Headache, as Long-Term Post-COVID Symptom After Severe Acute Respiratory SARS-CoV-2 Infection: A Case-Control Study. Front Hum Neurosci. 2021;15:678472
28. Planchuelo-Gómez Á, Trigo J, de Luis-García R, Guerrero ÁL, Porta-Etessam J, García-Azorín D. Deep Phenotyping of Headache in Hospitalized COVID-19 Patients via Principal Component Analysis. Front Neurol. 2020;11:583870.
29. Magdy R, Hussein M, Ragaie C, Abdel-Hamid HM, Khallaf A, Rizk HI, et al. Characteristics of headache attributed to COVID-19 infection and predictors of its frequency and intensity: A cross sectional study. Cephalalgia. 2020;40(13):1422–31.
30. Porta-Etessam J, Matías-Guiu JA, González-García N, et al. Spectrum of Headaches Associated With SARS-CoV-2 Infection: Study of Healthcare Professionals. Headache. 2020;60(8):1697-1704.
