# Supplementary material for: Headaches and facial pain attributed to SARS‐CoV‐2 infection and vaccination: a systematic review
Source: Eur J Neurol. 2024 Feb 28;31(6):e16251. doi: 10.1111/ene.16251 (PMC11235838; doi:10.1111/ene.16251)
Supplement: Supplementary file 3 — Appendix S3: [file ENE-31-e16251-s001.docx]

**Appendix No3**

**List of References for PICO 3**

1. Alfadda AA, Rafiullah M, Alkhowaiter M, et al. Clinical and biochemical characteristics of people experiencing post-coronavirus disease 2019-related symptoms: A prospective follow-up investigation. Front Med (Lausanne) 2022;9:1067082.

2. Bolay H, Karadas O, Ozturk B, et al. HMGB1, NLRP3, IL-6 and ACE2 levels are elevated in COVID-19 with headache: a window to the infection-related headache mechanism. J Headache Pain 2021;22:94.

3. Buzhdygan TP, DeOre BJ, Baldwin-Leclair A, et al. The SARS-CoV-2 spike protein alters barrier function in 2D static and 3D microfluidic in-vitro models of the human blood-brain barrier. Neurobiol Dis 2020;146:105131.

4. Caronna E, Ballve A, Llaurado A, et al. Headache: A striking prodromal and persistent symptom, predictive of COVID-19 clinical evolution. Cephalalgia 2020;40:1410-1421.

5. Caronna E, Alpuente A, Torres-Ferrus M, Pozo-Rosich P. Toward a better understanding of persistent headache after mild COVID-19: Three migraine-like yet distinct scenarios. Headache 2021;61:1277-1280.

6. Charfeddine S, Ibn Hadj Amor H, Jdidi J, et al. Long COVID 19 Syndrome: Is It Related to Microcirculation and Endothelial Dysfunction? Insights From TUN-EndCOV Study. Front Cardiovasc Med 2021;8:745758.

7. Charnley M, Islam S, Bindra GK, et al. Neurotoxic amyloidogenic peptides in the proteome of SARS-COV2: potential implications for neurological symptoms in COVID-19. Nat Commun 2022;13:3387.

8. Constant O, Barthelemy J, Bollore K, et al. SARS-CoV-2 Poorly Replicates in Cells of the Human Blood-Brain Barrier Without Associated Deleterious Effects. Front Immunol 2021;12:697329.

9. de Boni L, Odainic A, Gancarczyk N, et al. No serological evidence for neuronal damage or reactive gliosis in neuro-COVID-19 patients with long-term persistent headache. Neurol Res Pract 2022;4:53.

10. Dono F, Consoli S, Evangelista G, et al. New daily persistent headache after SARS-CoV-2 infection: a report of two cases. Neurol Sci 2021;42:3965-3968.

11. Espindola OM, Brandao CO, Gomes YCP, et al. Cerebrospinal fluid findings in neurological diseases associated with COVID-19 and insights into mechanisms of disease development. Int J Infect Dis 2021;102:155-162.

12. Fernandez-de-Las-Penas C, Gomez-Mayordomo V, Garcia-Azorin D, et al. Previous History of Migraine Is Associated With Fatigue, but Not Headache, as Long-Term Post-COVID Symptom After Severe Acute Respiratory SARS-CoV-2 Infection: A Case-Control Study. Front Hum Neurosci 2021;15:678472.

13. Garcia MA, Barreras PV, Lewis A, et al. Cerebrospinal fluid in COVID-19 neurological complications: Neuroaxonal damage, anti-SARS-Cov2 antibodies but no evidence of cytokine storm. J Neurol Sci 2021;427:117517.

14. Goshua G, Pine AB, Meizlish ML, et al. Endotheliopathy in COVID-19-associated coagulopathy: evidence from a single-centre, cross-sectional study. Lancet Haematol 2020;7:e575-e582.

15. Guilmot A, Maldonado Slootjes S, Sellimi A, et al. Immune-mediated neurological syndromes in SARS-CoV-2-infected patients. J Neurol 2021;268:751-757.

16. Jeong GU, Lyu J, Kim KD, et al. SARS-CoV-2 Infection of Microglia Elicits Proinflammatory Activation and Apoptotic Cell Death. Microbiol Spectr 2022;10:e0109122.

17. Karadas O, Ozturk B, Sonkaya AR, Tasdelen B, Ozge A, Bolay H. Latent class cluster analysis identified hidden headache phenotypes in COVID-19: impact of pulmonary infiltration and IL-6. Neurol Sci 2021;42:1665-1673.

18. Markovic SS, Gajovic N, Jurisevic M, et al. Galectin-1 as the new player in staging and prognosis of COVID-19. Sci Rep 2022;12:1272.

19. Meinhardt J, Radke J, Dittmayer C, et al. Olfactory transmucosal SARS-CoV-2 invasion as a port of central nervous system entry in individuals with COVID-19. Nat Neurosci 2021;24:168-175.

20. Planchuelo-Gomez A, Trigo J, de Luis-Garcia R, Guerrero AL, Porta-Etessam J, Garcia-Azorin D. Deep Phenotyping of Headache in Hospitalized COVID-19 Patients via Principal Component Analysis. Front Neurol 2020;11:583870.

21. Souza DD, Shivde S, Awatare P, et al. Headaches associated with acute SARS-CoV-2 infection: A prospective cross-sectional study. SAGE Open Med 2021;9:20503121211050227.

22. Straburzynski M, Nowaczewska M, Budrewicz S, Waliszewska-Prosol M. COVID-19-related headache and sinonasal inflammation: A longitudinal study analysing the role of acute rhinosinusitis and ICHD-3 classification difficulties in SARS-CoV-2 infection. Cephalalgia 2022;42:218-228.

23. Trigo J, Garcia-Azorin D, Planchuelo-Gomez A, et al. Factors associated with the presence of headache in hospitalized COVID-19 patients and impact on prognosis: a retrospective cohort study. J Headache Pain 2020;21:94.

24. Trigo J, Garcia-Azorin D, Sierra-Mencia A, et al. Cytokine and interleukin profile in patients with headache and COVID-19: A pilot, CASE-control, study on 104 patients. J Headache Pain 2021;22:51.

25. Tutal Gursoy G, Yuksel H, Mulkem Simsek I, et al. Neurological Presentations in Patients with COVID-19 in Cytokine Storm. Can J Neurol Sci 2023;50:89-95.

26. Uygun O, Ertas M, Ekizoglu E, et al. Headache characteristics in COVID-19 pandemic-a survey study. J Headache Pain 2020;21:121.

27. Virhammar J, Naas A, Fallmar D, et al. Biomarkers for central nervous system injury in cerebrospinal fluid are elevated in COVID-19 and associated with neurological symptoms and disease severity. Eur J Neurol 2021;28:3324-3331.
