# Supplementary material for: Headaches and facial pain attributed to SARS‐CoV‐2 infection and vaccination: a systematic review
Source: Eur J Neurol. 2024 Feb 28;31(6):e16251. doi: 10.1111/ene.16251 (PMC11235838; doi:10.1111/ene.16251)
Supplement: Supplementary file 4 — Appendix S4: [file ENE-31-e16251-s002.docx]

**Appendix No4**

**List of References for PICO 4**

1. Abukhalil AD, Shatat SS, Abushehadeh RR, Al-Shami N, Naseef HA, Rabba A. Side effects of Pfizer/BioNTech (BNT162b2) COVID-19 vaccine reported by the Birzeit University community. BMC Infect Dis 2023;23:5.

2. Albalawi OM, Alomran MI, Alsagri GM, Althunian TA, Alshammari TM. Analyzing the U.S. Post-marketing safety surveillance of COVID-19 vaccines. Saudi Pharm J 2022;30:180-184.

3. Alfaleh A, Alkattan A, Radwan N, et al. Adverse drug reactions from two COVID-19 vaccines reported in Saudi Arabia. Drugs Ther Perspect 2022;38:84-92.

4. Al-Hanawi MK, Keetile M, Kadasah NA, Alshareef N, Qattan AMN, Alsharqi O. Side Effects and Perceptions of COVID-19 Vaccination in Saudi Arabia: A Cross-Sectional Study. Front Med (Lausanne) 2022;9:899517.

5. Alharbi NK, Al-Tawfiq JA, Alghnam S, et al. Outcomes of single dose COVID-19 vaccines: Eight month follow-up of a large cohort in Saudi Arabia. J Infect Public Health 2022;15:573-577.

6. Almughais ES, Alharbi AH, Aldarwish HA, et al. Side-effects of COVID-19 vaccines among the Saudi population: A cross-sectional study. Saudi Med J 2022;43:386-393.

7. Amaro C, Monteiro C, Duarte AP. COVID-19 Vaccines Adverse Reactions Reported to the Pharmacovigilance Unit of Beira Interior in Portugal. J Clin Med 2022;11.

8. Babamahmoodi F, Saeedi M, Alizadeh-Navaei R, et al. Side effects and Immunogenicity following administration of the Sputnik V COVID-19 vaccine in health care workers in Iran. Sci Rep 2021;11:21464.

9. Baldolli A, Michon J, Appia F, Galimard C, Verdon R, Parienti JJ. Tolerance of BNT162b2 mRNA COVI-19 vaccine in patients with a medical history of COVID-19 disease: A case control study. Vaccine 2021;39:4410-4413.

10. Ballestero M, Souza RLP, Sakae TM, Costa L, Furlanetti L, Oliveira RS. Post-vaccination incidence and side effects of COVID-19 in a cohort of Brazilian healthcare professionals: an internet-based survey. Einstein (Sao Paulo) 2022;20:eAO0067.

11. Baydar O, Ozen S, Ozturk Sahin B, Kokturk N, Kitapci MT. Safety of an Inactivated SARS-CoV-2 Vaccine Among Healthcare Workers in Turkey: An Online Survey. Balkan Med J 2022;39:193-198.

12. Borroni E, Consonni D, Cugno M, et al. Side effects among healthcare workers from a large Milan university hospital after second dose of BNT162b2 mRNA COVID-19 vaccine. Med Lav 2021;112:477-485.

13. Canas LS, Osterdahl MF, Deng J, et al. Disentangling post-vaccination symptoms from early COVID-19. EClinicalMedicine 2021;42:101212.

14. Cuschieri S, Borg M, Agius S, Souness J, Brincat A, Grech V. Adverse reactions to Pfizer-BioNTech vaccination of healthcare workers at Malta's state hospital. Int J Clin Pract 2021;75:e14605.

15. Deng L, Glover C, Dymock M, et al. The short term safety of COVID-19 vaccines in Australia: AusVaxSafety active surveillance, February - August 2021. Med J Aust 2022;217:195-202.

16. Di Valerio Z, La Fauci G, Solda G, et al. ROCCA cohort study: Nationwide results on safety of Gam-COVID-Vac vaccine (Sputnik V) in the Republic of San Marino using active surveillance. EClinicalMedicine 2022;49:101468.

17. Dreyer N, Reynolds MW, Albert L, et al. How frequent are acute reactions to COVID-19 vaccination and who is at risk? Vaccine 2022;40:1904-1912.

18. Dutta S, Kaur R, Charan J, et al. Analysis of Neurological Adverse Events Reported in VigiBase From COVID-19 Vaccines. Cureus 2022;14:e21376.

19. Ekizoglu E, Gezegen H, Yalinay Dikmen P, Orhan EK, Ertas M, Baykan B. The characteristics of COVID-19 vaccine-related headache: Clues gathered from the healthcare personnel in the pandemic. Cephalalgia 2022;42:366-375.

20. Farhat M, Al-Ibrahim R, Almohammedali A, Aljishi R, Alalwan B. Study of the Side Effects of Pfizer and Oxford COVID-19 Vaccines in the Eastern Province of Saudi Arabia. Int J Gen Med 2022;15:7547-7558.

21. Ganesan S, Al Ketbi LMB, Al Kaabi N, et al. Vaccine Side Effects Following COVID-19 Vaccination Among the Residents of the UAE-An Observational Study. Front Public Health 2022;10:876336.

22. Guzel EC, Yildiz T, Buyukkiyici O, Dombaz OE. Safety and Adverse Effects of Inactive SARS-Cov-2 Vaccine (CoronaVac) in Health Care Professionals. J Pak Med Assoc 2022;72:1792-1796.

23. Halperin SA, Ye L, MacKinnon-Cameron D, et al. Final efficacy analysis, interim safety analysis, and immunogenicity of a single dose of recombinant novel coronavirus vaccine (adenovirus type 5 vector) in adults 18 years and older: an international, multicentre, randomised, double-blinded, placebo-controlled phase 3 trial. Lancet 2022;399:237-248.

24. Hatmal MM, Al-Hatamleh MAI, Olaimat AN, et al. Reported Adverse Effects and Attitudes among Arab Populations Following COVID-19 Vaccination: A Large-Scale Multinational Study Implementing Machine Learning Tools in Predicting Post-Vaccination Adverse Effects Based on Predisposing Factors. Vaccines (Basel) 2022;10.

25. Im JH, Kim E, Lee E, et al. Adverse Events with the Pfizer-BioNTech COVID-19 Vaccine among Korean Healthcare Workers. Yonsei Med J 2021;62:1162-1168.

26. Kadali RAK, Janagama R, Peruru S, Malayala SV. Side effects of BNT162b2 mRNA COVID-19 vaccine: A randomized, cross-sectional study with detailed self-reported symptoms from healthcare workers. Int J Infect Dis 2021;106:376-381.

27. Kaur S, Singh A, Saini S, et al. Reporting adverse events of ChAdOx1 nCoV-19 coronavirus vaccine (Recombinant) among the vaccinated healthcare professionals: A cross-sectional survey. Indian J Med Res 2022;155:123-128.

28. Kitagawa H, Kaiki Y, Sugiyama A, et al. Adverse reactions to the BNT162b2 and mRNA-1273 mRNA COVID-19 vaccines in Japan. J Infect Chemother 2022;28:576-581.

29. Konu YR, Gbeasor-Komlanvi FA, Yerima M, et al. Prevalence of severe adverse events among health professionals after receiving the first dose of the ChAdOx1 nCoV-19 coronavirus vaccine (Covishield) in Togo, March 2021. Arch Public Health 2021;79:207.

30. Kremsner PG, Ahuad Guerrero RA, Arana-Arri E, et al. Efficacy and safety of the CVnCoV SARS-CoV-2 mRNA vaccine candidate in ten countries in Europe and Latin America (HERALD): a randomised, observer-blinded, placebo-controlled, phase 2b/3 trial. Lancet Infect Dis 2022;22:329-340.

31. Kuodi P, Gorelik Y, Zayyad H, et al. Association between BNT162b2 vaccination and reported incidence of post-COVID-19 symptoms: cross-sectional study 2020-21, Israel. NPJ Vaccines 2022;7:101.

32. Magdy R, Khedr D, Yacoub O, Attia A, Abdelrahman MA, Mekkawy D. Epidemiological aspects of headache after different types of COVID-19 vaccines: An online survey. Headache 2022;62:1046-1052.

33. Makadzange AT, Gundidza P, Lau C, et al. Vaccine Adverse Events Following COVID-19 Vaccination with Inactivated Vaccines in Zimbabwe. Vaccines (Basel) 2022;10.

34. Martins-Filho PR, Santana RRR, Cavalcante TF, et al. Surveillance of adverse events associated with 145 000 doses of COVID-19 vaccines in a Brazilian municipality. Rev Panam Salud Publica 2022;46:e110.

35. McMurry R, Lenehan P, Awasthi S, et al. Real-time analysis of a mass vaccination effort confirms the safety of FDA-authorized mRNA COVID-19 vaccines. Med 2021;2:965-978 e965.

36. Montalti M, Solda G, Di Valerio Z, et al. ROCCA observational study: Early results on safety of Sputnik V vaccine (Gam-COVID-Vac) in the Republic of San Marino using active surveillance. EClinicalMedicine 2021;38:101027.

37. Naito T, Tsuchida N, Kusunoki S, et al. Reactogenicity and immunogenicity of BNT162b2 or mRNA-1273 COVID-19 booster vaccinations after two doses of BNT162b2 among healthcare workers in Japan: a prospective observational study. Expert Rev Vaccines 2022;21:1319-1329.

38. Nassar RI, Alnatour D, Thiab S, Nassar A, El-Hajji F, Basheti IA. Short-term side effects of COVID-19 vaccines: A cross-sectional study in Jordan. Hum Vaccin Immunother 2022;18:2082792.

39. Niesen MJM, Pawlowski C, O'Horo JC, et al. Surveillance of Safety of 3 Doses of COVID-19 mRNA Vaccination Using Electronic Health Records. JAMA Netw Open 2022;5:e227038.

40. Oh HK, Kim EK, Hwang I, et al. COVID-19 vaccine safety monitoring in the Republic of Korea: February 26, 2021 to April 30, 2021. Osong Public Health Res Perspect 2021;12:264-268.

41. Oleszczyk M, Marciniak Z, Nessler K, et al. COVID-19 vaccine short-term adverse events in the real-life family practice in Krakow, Poland. Eur J Gen Pract 2023;29:2147500.

42. Omeish H, Najadat A, Al-Azzam S, et al. Reported COVID-19 vaccines side effects among Jordanian population: a cross sectional study. Hum Vaccin Immunother 2022;18:1981086.

43. Orebi HA, Emara HE, Alhindi AA, et al. Perceptions and experiences of COVID-19 vaccines' side effects among healthcare workers at an Egyptian University Hospital: a cross-sectional study. Trop Med Health 2022;50:37.

44. Ortiz-Prado E, Izquierdo-Condoy JS, Fernandez-Naranjo R, et al. A Comparative Analysis of a Self-Reported Adverse Events Analysis after Receiving One of the Available SARS-CoV-2 Vaccine Schemes in Ecuador. Vaccines (Basel) 2022;10.

45. Polack FP, Thomas SJ, Kitchin N, et al. Safety and Efficacy of the BNT162b2 mRNA Covid-19 Vaccine. N Engl J Med 2020;383:2603-2615.

46. Rivera-Izquierdo M, Soler-Iborte E, de Rojas JP, et al. Factors Associated with Adverse Reactions to BNT162b2 COVID-19 Vaccine in a Cohort of 3969 Hospital Workers. Vaccines (Basel) 2021;10.

47. Rosenblum HG, Gee J, Liu R, et al. Safety of mRNA vaccines administered during the initial 6 months of the US COVID-19 vaccination programme: an observational study of reports to the Vaccine Adverse Event Reporting System and v-safe. Lancet Infect Dis 2022;22:802-812.

48. Saeed BQ, Al-Shahrabi R, Alhaj SS, Alkokhardi ZM, Adrees AO. Side effects and perceptions following Sinopharm COVID-19 vaccination. Int J Infect Dis 2021;111:219-226.

49. Saita M, Yan Y, Ito K, Sasano H, Seyama K, Naito T. Reactogenicity following two doses of the BNT162b2 mRNA COVID-19 vaccine: Real-world evidence from healthcare workers in Japan. J Infect Chemother 2022;28:116-119.

50. Shapiro Ben David S, Baruch Gez S, Rahamim-Cohen D, Shamir-Stein N, Lerner U, Ekka Zohar A. Immediate side effects of Comirnaty COVID-19 vaccine: A nationwide survey of vaccinated people in Israel, December 2020 to March 2021. Euro Surveill 2022;27.

51. Shenoy ES, Wickner PG, West LR, et al. Symptom monitoring after coronavirus disease 2019 (COVID-19) vaccination in a large integrated healthcare system: Separating symptoms from severe acute respiratory coronavirus virus 2 (SARS-CoV-2) infection. Infect Control Hosp Epidemiol 2022;43:1439-1446.

52. Shimamura Y, Anbo Y, Furuta Y. Post-vaccination Adverse Reactions After Receiving the Pfizer-BioNTech Coronavirus Disease 2019 Vaccines Among Healthcare Workers in Sapporo, Japan. Cureus 2022;14:e23549.

53. Song JY, Cheong HJ, Kim SR, et al. Early Safety Monitoring of COVID-19 Vaccines in Healthcare Workers. J Korean Med Sci 2021;36:e110.

54. Spyker DA, Bronstein AC, Weber JA. Making US poison centers a part of the solution to the COVID-19 pandemic. Clin Toxicol (Phila) 2022;60:102-114.

55. Takuva S, Takalani A, Seocharan I, et al. Safety evaluation of the single-dose Ad26.COV2.S vaccine among healthcare workers in the Sisonke study in South Africa: A phase 3b implementation trial. PLoS Med 2022;19:e1004024.

56. Tosun S, Ozkan Ozdemir H, Erdogan E, et al. Adverse events report of inactivated COVID-19 vaccine from 4040 healthcare workers. Postgrad Med 2022;134:104-110.

57. Tran VN, Nguyen HA, Le TTA, Truong TT, Nguyen PT, Nguyen TTH. Factors influencing adverse events following immunization with AZD1222 in Vietnamese adults during first half of 2021. Vaccine 2021;39:6485-6491.

58. Undugodage C, Dissanayake U, Kumara H, et al. Reactogenicity to ChAdOx1 nCoV-19 vaccine in health care workers: A multicenter observational study in Sri Lanka. Ceylon Med J 2021;66:177-184.

59. Vigezzi GP, Lume A, Minerva M, et al. Safety surveillance after BNT162b2 mRNA COVID-19 vaccination: results from a cross-sectional survey among staff of a large Italian teaching hospital. Acta Biomed 2021;92:e2021450.

60. Wang XY, Mahmood SF, Jin F, et al. Efficacy of heterologous boosting against SARS-CoV-2 using a recombinant interferon-armed fusion protein vaccine (V-01): a randomized, double-blind and placebo-controlled phase III trial. Emerg Microbes Infect 2022;11:1910-1919.

61. Yih WK, Daley MF, Duffy J, et al. A broad assessment of covid-19 vaccine safety using tree-based data-mining in the vaccine safety datalink. Vaccine 2023;41:826-835.

62. Zhang MX, Zhang TT, Shi GF, et al. Safety of an inactivated SARS-CoV-2 vaccine among healthcare workers in China. Expert Rev Vaccines 2021;20:891-898.
