# Supplementary material for: Headaches and facial pain attributed to SARS‐CoV‐2 infection and vaccination: a systematic review
Source: Eur J Neurol. 2024 Feb 28;31(6):e16251. doi: 10.1111/ene.16251 (PMC11235838; doi:10.1111/ene.16251)
Supplement: Supplementary file 5 — Appendix S5: [file ENE-31-e16251-s004.docx]

**Appendix No5**

**List of references for PICO 5**

1. Ekizoglu E, Gezegen H, Yalınay Dikmen P, Orhan EK, Ertaş M, Baykan B. The characteristics of COVID-19 vaccine-related headache: Clues gathered from the healthcare personnel in the pandemic. Cephalalgia [Internet]. 2022 Apr 1 [cited 2023 May 17];42(4–5):366–75. Available from: https://pubmed.ncbi.nlm.nih.gov/34510919/
2. Göbel CH, Heinze A, Karstedt S, Morscheck M, Tashiro L, Cirkel A, et al. Headache Attributed to Vaccination Against COVID-19 (Coronavirus SARS-CoV-2) with the ChAdOx1 nCoV-19 (AZD1222) Vaccine: A Multicenter Observational Cohort Study. Pain Ther [Internet]. 2021 Dec 1 [cited 2023 May 17];10(2):1309–30. Available from: https://pubmed.ncbi.nlm.nih.gov/34313952/
3. Göbel CH, Heinze A, Karstedt S, Morscheck M, Tashiro L, Cirkel A, et al. Clinical characteristics of headache after vaccination against COVID-19 (coronavirus SARS-CoV-2) with the BNT162b2 mRNA vaccine: a multicentre observational cohort study. Brain Commun [Internet]. 2021 [cited 2023 May 17];3(3). Available from: https://pubmed.ncbi.nlm.nih.gov/34405142/
4. Ceccardi G, Schiano di Cola F, Di Cesare M, Liberini P, Magoni M, Perani C, et al. Post COVID-19 vaccination headache: A clinical and epidemiological evaluation. Frontiers in pain research (Lausanne, Switzerland) [Internet]. 2022 Nov 8 [cited 2023 May 17];3. Available from: https://pubmed.ncbi.nlm.nih.gov/36425358/
